# Bridging the gap in fishing effort mapping: a spatially-explicit fisheries dataset for Campanian MPAs, Italy

**DOI:** 10.1038/s41597-023-02883-9

**Published:** 2024-01-09

**Authors:** Pamela Lattanzi, Jacopo Pulcinella, Pietro Battaglia, Antonio Di Cintio, Carmen Ferrà, Antonio Di Franco, Anna Nora Tassetti

**Affiliations:** 1 National Research Council, Institute of Marine Biological Resources and Biotechnologies (CNR-IRBIM), Ancona, Italy; 2 Department of Biological, Geological and Environmental Sciences (BiGeA), University of Bologna, Bologna, Italy; 3 NBFC, National Biodiversity Future Center, Palermo, Italy; 4 Stazione Zoologica Anton Dohrn, National Institute of Biology, Ecology and Marine Biotechnology, Sicily Marine Centre, Messina, Italy; 5 Stazione Zoologica Anton Dohrn, National Institute of Biology, Ecology and Marine Biotechnology, Villa Comunale, Naples, Italy; 6 Stazione Zoologica Anton Dohrn, National Institute of Biology, Ecology and Marine Biotechnology, Sicily Marine Centre, Palermo, Italy

**Keywords:** Ecosystem ecology, Conservation biology, Biodiversity, Water resources, Marine biology

## Abstract

Recent technological advancements have facilitated the extensive collection of movement data from large-scale fishing vessels, yet a significant data gap remains for small-scale fisheries. This gap hinders the development of consistent exploitation patterns and meeting the information needs for marine spatial planning in fisheries management. This challenge is specifically addressed in the Campania region of Italy, where several Marine Protected Areas support biodiversity conservation and fisheries management. The authors have created a spatially-explicit dataset that encompasses both large-scale (vessels exceeding 12 meters in length) and small-scale (below 12 meters) fishing efforts. This dataset (available at 10.6084/m9.figshare.23592006) is derived from vessel tracking data and participatory mapping. It offers insights into potential conflicts between different fishing segments and their interactions with priority species and habitats. The data can assist researchers and coastal management stakeholders in formulating policies that reduce resource competition and promote ecosystem-based fisheries management. Furthermore, the provided mapping approach is adaptable for other regions and decision-making frameworks, as we are committed to sharing the tools and techniques we employed.

## Background & Summary

Marine Spatial Planning (MSP) is being widely implemented as a public process of analysing the spatiotemporal distribution of human activities in marine areas to achieve economic, environmental and social objectives that are usually specified through a political process. It is conceived to spatially allocate and manage maritime human activities while reducing inter-sectoral conflicts and environmental impacts, and promoting the sustainable use of marine ecosystems^1^. Adopting a decision-making framework based on spatially-explicit data, MSP seeks to advance ecosystem-based sea use management^2,3^, drawing up plans for identifying the utilisation of maritime space for different sea uses^4^.

Indeed, being able to table detailed and robust spatial quantitative data is key in many MSP processes, and could become critical when dealing with fisheries and related spatially explicit knowledge at proper scales^5^.

In fact, even though there is a large consensus about the need to factor fisheries data into MSP assessments^5–8^, its actual integration often fails due to various challenges^9^, such as identifying where fishing effort is actually allocated, which drivers affect fishers’ behaviour, and where commercial fish species seasonally and spatially move under various anthropogenic pressures and along their successive life stages.

Actually, an overwhelming amount of spatiotemporal information on industrial/large-scale fisheries (LSF) and related movements is made available by various non-cooperative remote sensing systems and ship reporting technologies^10^. These include Vessel Monitoring System (VMS, mandatory with Electronic logbook for all European fishing vessels in excess of 12 m LOA from 1 January 2012), and Automatic Identification System (AIS, required to be carried on board of EU fishing vessels longer than 15 m^11^). From an MSP perspective, this latter has become widely popular and available, even though it was originally conceived as a collision-avoidance tool for European fishing vessels above 15 m in length (EU Dir 2011/15/EU). Indeed, it has been increasingly used to track fishing vessels and estimate fishing pressure^12^, map exploited fishing grounds^13,14^ and support decision-making processes^5^, as well as to rate the effectiveness of spatial management measures and the presence of unauthorised fishing^15–19^.

The other side of the coin is represented by data-poor small-scale fisheries (SSF; i.e., vessels under 12 m in length and not using towed fishing gears, as defined in the CE Regulation N° 508/2014).

However, nowadays the important role played by SSFs from a biological, economic and societal perspective is finally acknowledged and this is particularly significant in the Mediterranean Sea, where SSFs make up 82% of the total fleet, providing 59% of total onboard employment, 27% of total revenue and 15% of the total catch^20^. The growing awareness concerning the possible impacts of SSFs on stocks has led the European Union to set up policies for the traceability of small-scale vessels’ movements by all the Member States, as a means to control their fishing activities in compliance with the rules of the Common Fisheries Policy^21^. Having recognized this historical information gap, experts and authorities are stepping up their efforts to encourage SSF data collection on a local/regional scale^22–24^. This is the case of the General Fisheries Commission for the Mediterranean (GFCM), which launched the *Regional Plan of Action for Small-Scale Fisheries in the Mediterranean and the Black Sea* (RPOA-SSF) to undertake concrete actions up to 2028 to strengthen and support sustainable small-scale fishing^23^. Furthermore, the International Council for the Exploration of the Sea (ICES) have recently organised working groups specifically aiming at discussing methods for working with high-resolution geospatial data in small-scale fisheries and mapping the relative fishing effort^25,26^.

Therefore, many countries are recently pushing for the exploitation of different tracking technologies (e.g., AIS^27^, VMS^28^, GNSS^29^) to get this kind of information and for the development of several procedures aimed at categorizing fishing boats’ behaviour and furnishing quantitative approaches for SSF management^30,31^.

Today, even in some developing nations, a certain amount of pilot studies gathering data from low-cost GPS tracking devices have been carried out, trying to figure out (sometimes with the help of modelling techniques) the spatiotemporal extent of local fisheries as a way to support the resource management and the precious societal-economic role of this sector^32–34^.

Nevertheless, the collection of SSFs positional information is still not mandatory and the absence of an official legislative regulation regarding data collection further hampers the overall understanding of the activity and the spatial dynamic of this fleet segment^27,34–37^. Indeed, the challenge of managing marine resources is often hindered in SSFs data-poor scenarios. However, this situation can be improved through cost-effective approaches which contribute to generate informed baseline data for decision-making. For instance, participatory methods allow researchers to produce maps which serve as effective visualisation tools facilitating communication and interaction among stakeholders^38^. Furthermore, collecting SSFs data through the active involvement of fishers is a priority requirement even in the context of the RPOA-SSF, in order to integrate fisher Local Ecological Knowledge (LEK) in management plans^23^.

Even if something is changing^39^, the limited (in time and space) majority of the spatially explicit information we have today on Italian SSF mainly relies on participatory approaches, such as those promoted by international cooperation programs like *AdriaMed* and *MedSudMed*^40^.

Here we provide fishing effort data of large- (LOA >12 m) and small-scale (LOA <12 m) vessels operating in the Campania region (Italy), relying on AIS (poll frequency of 5 minutes) to observe patterns of LSF trawling and purse-seining activities and on participatory mapping to spatially characterise SSF effort and related targeted species. Supplying concrete layers of information, the present work consolidates the knowledge framework on the fishing sector of the Campania region and supports management measures such as those in force in its Marine Protected Areas (MPAs)^41^.

We envisage that the fishing effort data provided here will be of great use to local researchers and administrators who seek to understand the interactions between multiple stressors and their impacts on coastal marine resources and the environment. Furthermore, it will help them set up decisions that could support small-scale fishers’ incomes and well-being while enhancing fisheries’ sustainability^5,9,42–45^.

Sharing codes and instruments together with the effort data (10.6084/m9.figshare.23592006^46^), additional value is given to the proposed methodological workflow as readers could be able to tailor and reproduce it in other contexts and decision-making frameworks.

## Methods

**Fig. 1:**
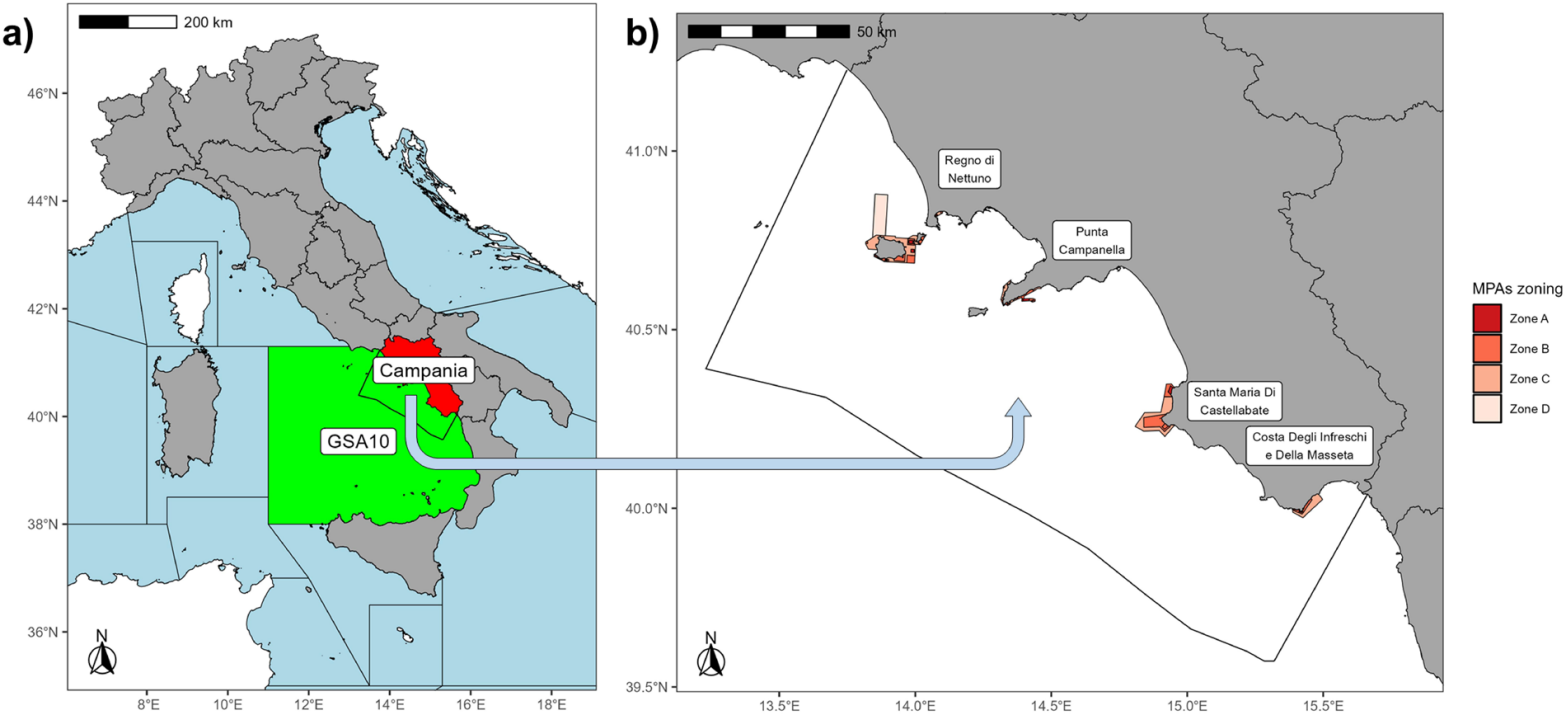
Study area (surrounded by a black border) within GSA10 **(a)** and the 4 Campanian MPAs **(b)**. Different zones, associated with different allowed human uses, are indicated per each MPA.

The study area covers the Campania region (Fig. 1), which is located in the Geographical Sub-Area 10 (GSA10 - Southern and Central Tyrrhenian Sea) with a coastline of 502 km (6% of the Italian coast).

The Region has a deep-rooted fishing tradition and maintains a fishing fleet comprising five primary categories: trawlers, purse seiners, hydraulic dredgers, small-scale vessels, and large-scale polyvalent passive vessels. Significantly, SSFs dominate the region, making up 85% of the local fleet, as opposed to the national average of 68%^47^. These vessels typically operate in the Region’s coastal waters, as well as within or near the four established Marine Protected Areas (MPAs) known as *Regno di Nettuno, Punta Campanella, Santa Maria di Castellabate*, and *Costa degli Infreschi e della Masseta*^48–50^.

It must be kept in mind that a zoning strategy with different levels of protection is here applied: in zone A, any form of fishing is prohibited; in zones B and C SSF is generally permitted and regulated; while in zone D (which occurs only in the *Regno di Nettuno* case) LSF is also authorized.

For processing purposes, the study area was downloaded from the geoportal (https://geonetwork.bioinfo.szn.it/geonetwork/) built in the framework of the same project (ISSPA - Innovation, development and sustainability for the fisheries and aquaculture sectors in the Campania region, PO FEAMP 2014/2020, Mis. 1.40) funded by the European Maritime and Fisheries Fund, as well as the spatial layers of the Campanian MPAs.

**Fig. 2:**
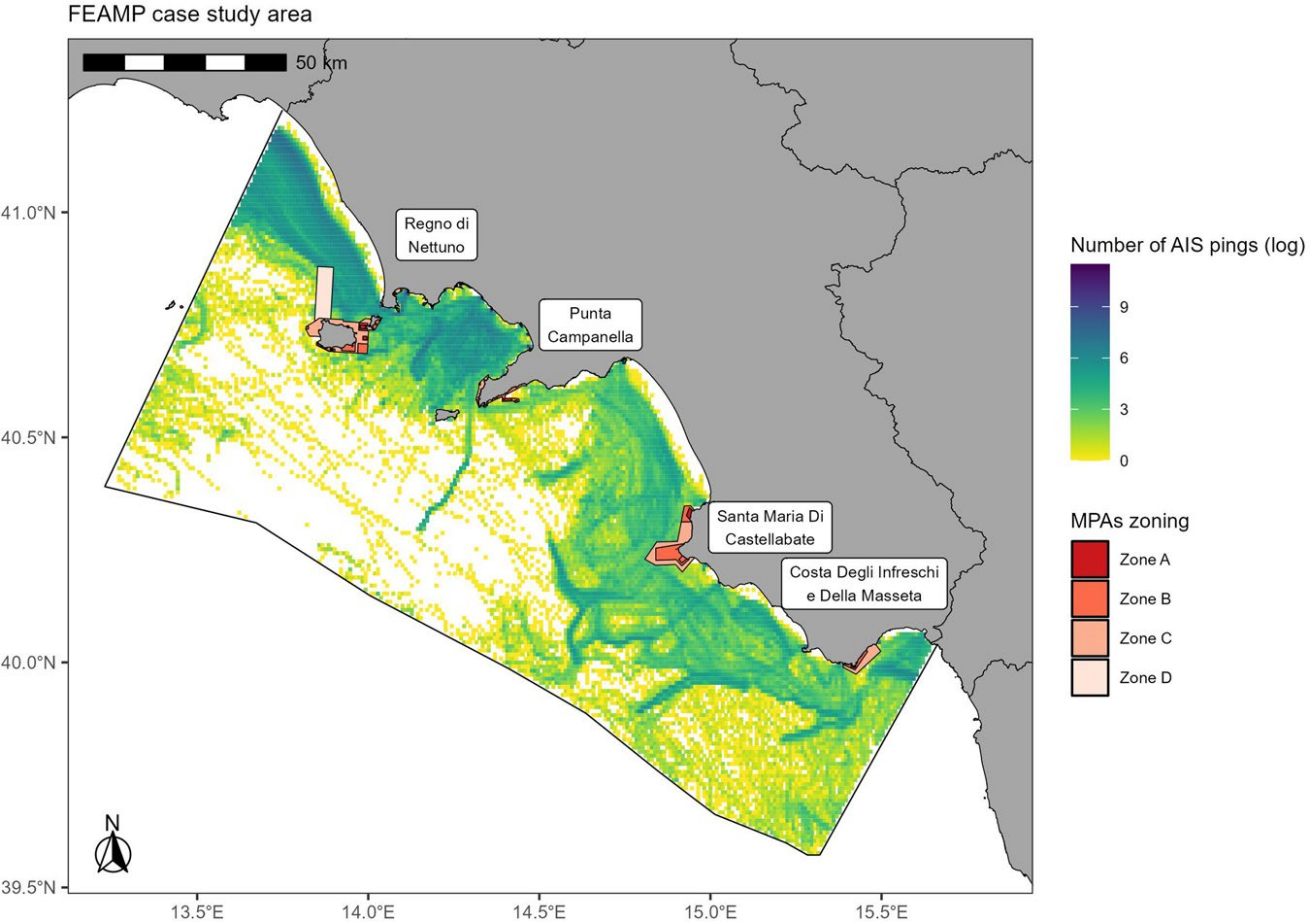
AIS broadcasts by LSFs (density in each 0.01° × 0.01°–1 km × 1 km - grid cell, year 2019) and MPAs zoning (i.e., locations of the SSF participatory mapping).

Spatially-explicit information on large- and small-scale fishing effort was integrated, combining vessel tracking data processing and participatory mapping within the four nationally-designated MPAs located in the study area (Fig. 2).

Industrial fishing pressure was estimated in terms of annual and monthly fishing hours, by processing terrestrial AIS data (year 2019, poll frequency of 5 min). R scripts are available in the R4AIS code repository v1.0.2^51^ (https://github.com/MAPSirbim/AIS_data_processing/tree/v1.0.2, last access: 28 June 2023). Released data were gridded at 0.05° × 0.05° (5 km × 5 km) resolution in GSA10, and at 0.01° × 0.01° (1 km × 1 km) resolution within the MPAs case study area. A quantitative evaluation of unobserved fishing activity was also given by considering traces of signal absence (exceeding 30 min), which could be attributed to system malfunction (e.g., in areas of high maritime traffic) or an intentional shutdown by fishers^52^.

Preferring AIS over VMS data primarily stemmed from their accessibility, as they could be procured affordably from private providers^53^. These data were well-suited for the detailed mapping of fishing activities due to their high spatial and temporal precision^53^ because, contrary to VMS data, given the high frequency of transmission of the signal, AIS data do not require to be interpolated.

On the other side, due to the lack of spatially-explicit data for the Campanian SSF segment (LOA < 12 m), a participatory approach was chosen for characterising it and mapping its fishing grounds within and around the MPAs. SSF data were collected through 71 individual semi-structured face-to-face interviews with fishers (i.e. one per fishing boat), which do not revolve around fishing trips, but instead focus on exploring the typical fishing locations that each fisher frequents. Each fisher sketched out the specific areas they target with their fishing gear and provided information on the number of months they engage in fishing activities in 2019 for each type of gear. Furthermore, each fisher detailed the precise number of fishing days per each month.

An interactive web app was developed and used to digitise the spatially-explicit answers. SSF digitised polygons were processed in QGIS and fishing pressure was aggregated at 0.01° × 0.01° (1 km × 1 km) resolution and given in terms of monthly and annual fishing days by employed gear. Gridding at the same 0.01° × 0.01° (1 km × 1 km) resolution within the MPAs case study area allowed authors to integrate AIS outcome and bridge a solid informative layer, which is key for MSP and integrated management of coastal areas.

Although fishing within MPAs is mainly practised by small-scale vessels (as in most cases MPAs allow only small-scale professional fishing, with few exceptions as *Regno di Nettuno*), the analysis of the activity carried out by AIS-equipped larger vessels contributed to outlining a more detailed and integrated pressure scenario close to the MPAs. Their extension and the information relating to their zoning were used in the ISSPA project to verify the presence of fishing activities in these areas.

### Large-scale fisheries - AIS data processing

The analysis was based on the terrestrial AIS of year 2019, owned by CNR-IRBIM and obtained from a private provider (http://www.astrapaging.com/, last access: 1 July 2023), with a poll frequency of 5 min and including European Union (EU) and non-EU vessels (AIS type = 30, “Fishing”). Data is hosted in a local spatial-type relational database (Postgres with Postgis extension) that is continuously maintained and updated by the owner, and is structured according to the published R4AIS workflow^51^. Historical raw broadcasts were imported as spatial features (i.e., point geometry data type) in vessel-specific tables, and processed by applying the R scripts made available by the R4AIS code repository^51^ in order to (i) reconstruct individual fishing trips, (ii) classify them on a monthly basis according to predefined gear classes, and (iii) infer fishing operations, such as tracks of trawlers (while deploying the gear) and centroids of the drifting clusters that purse seiners designed while hauling in the seine net.

In particular, the workflow includes the identification of individual fishing trips, that is the sequence of points describing a single harbour-to-harbour movement of a vessel. The fishing trips’ identification requires the processing of the data in order to identify the positions in the harbour and to split the data according to the events of entry and exit from a harbour.

Once vessels’ raw data were divided into fishing trips, they were classified in order to identify the fishing activity of the vessels (i.e. the gear used). The R4AIS method^51^ involves the application of a semi-automatic classifier which takes into account position, speed and depth to classify fishing trips according to predefined gear classes.

Finally, the R4AIS workflow^51^ involves the identification of positions of fishing operations. Depending on the type of gear, fishing operations were stored in different types of geometries, which reflect the different spatial behaviour of each fishing gear (or group of similar gears). For example, fishing positions of towed gear, such as OTB, TBB or PTM, are stored as line geometries, because they describe the path taken with the gear deployed. On the contrary, the fishing positions of purse-seiners are stored as points, which describe the centroids of the drifting clusters that the vessel designed while hauling the seine net. Processing outcomes, such as reconstructed trips and inferred fishing segments/points, were stored as linestring/point geometries in related vessel-specific tables.

The spatial layer of fishing effort activity of the LSF fleet provided in the present work was created from our AIS database. The analyzed dataset contains all fishing geometries, identified during the processing steps, that occurred at least once in 2019 within the GSA10 and the MPAs case study area. The fishing activity in each grid cell was then estimated by (i) intersecting retrieved fishing tracks/points with the grid, (ii, only for trawlers) computing the durations of each trawling portion overlapping the cell (i.e., dividing the resulting length by the inherited mean fishing speed that is stored in the fishing segments’ table), and (iii) aggregating fishing hours both by month and by year.

Within the study area, the possible presence of “hidden” fishing activity was also quantified according to the approach described by Ferrà *et al*.^52^. Vessel tracks were analyzed under the assumption that they follow straight-line paths. The duration of each track segment and the speed of the vessels were determined by calculating the differences between consecutive location pings. Given a reporting rate of every 5 minutes, any tracks with a duration exceeding a predetermined threshold of 30 minutes were classified as “unknown”. These tracks were kept and treated as instances of transmission gaps. To determine if certain gaps, located in the main fishing areas, might be concealing fishing operations, the authors examined the unidentified track segments that overlapped with the hotspots identified in the trawl footprint^12^. Those located within harbours were disregarded. A subset of unknown segments was generated selecting the duration and speed values that occurred most frequently. Following validation, considering the typical speed range of bottom trawlers in the Mediterranean Sea (2–5 knots), these segments were categorized as potential indicators of hidden fishing activity. As well as the AIS-observed, the hidden fishing activity, quantified as fishing hours, was additionally aggregated yearly.

To identify the active fleet at the time of the analyses, it was necessary to link the information from the official online database of fleet registers with the Campanian regional fishing fleet register (named “FEAMP register”), considered as the main reference register because it was supposed to be more updated than the others. The official registers that were checked with the main reference are: the European Fleet Register^54^ (tracking all the events in the history of European vessels, and available at: https://webgate.ec.europa.eu/fleet-europa/index_en, last access: 23 July 2021), and the GFCM fleet register (transmitted by Contracting Parties and Cooperating non-contracting Parties to the GFCM Secretariat, and available at: http://www.fao.org/gfcm/data/fleet/register/en/, last access: 23 July 2021). The latter represents the equivalent of the European Fleet Register for all fishing vessels operating in the Mediterranean, but belonging to nations not part of the European Union.

Official registers, tracking all events that occurred in the history of a vessel, allow the enrichment of the dataset with administrative information, which in turn can be used to improve the processing of the data and results of the analysis. For example, the reallocation of vessels due to a change in the place of registration, as well as events of license changes or decommissioning can be used to identify temporal change of local fleets in terms of the number of vessels, divided by fishing gears and/or fleet segments.

A step-by-step matching procedure was developed to progressively link the AIS data subset with all the available fleet registers. The matching was performed to link pairs of records based on different keys (i.e., MMSI = Maritime Mobile Service Identity, IRCS = International Radio Call Sign, NAME = vessel name, and IMO = International Maritime Organization identification number). In order to solve problems of misspelling, matching between fields was quantified using the Levenshtein strings distance function (R package “stringdist”^55^), which counts the number of deletions, insertions and substitutions necessary to turn one string into the other and calculates the degree of the pairwise string alignment. In each step, a field was used as a primary key while a secondary field was used to cross-check the match, which was considered successful if the primary key was accurate (distance = 0) and the distance between the secondary field was below a specified threshold (distance < 3). The NAME was used as the secondary field when records were first matched by MMSI (and by IMO and IRCS in the following steps); while IRCS was used to cross-check the last joining by NAME. At each step, unpaired records were manually checked before being used as input for the following matching in the cascade, and finally before proceeding to the next register.

As a result of the matching process, AIS data were uniquely associated with their gear licences (primary and secondary licences), flag country, registration site, and overall length (LOA). In particular, vessels authorised to only one type of fishing (therefore without a secondary fishing licence) were used to strengthen the training of the classification algorithms and validate the classification results, while the LOA was used to categorise AIS data into 4 main vessel length (VL) classes: VL(1218], VL(1824], VL(2440] and VL(40XX). However, since changes between licensed fishing gears are not accounted for in the official registers because they occur quickly and for short periods, for vessels with multiple licenses we used the classification model to capture the changes in fishing behaviour (i.e. fishing gear) that occurred during the study period.

### Small-scale fisheries - Participatory mapping

A participatory mapping approach was employed based on the methodology previously tested by Grati *et al*.^40^ (see Supplementary Material S1 for an example of the interviews carried out). Spatially explicit SSF information was collected utilising pre-print maps of the coastal areas covering each MPA and its surroundings. Maps were (i) produced using background map tiles from OpenStreetMap (http://tile.openstreetmap.org/{z}/{x}/{y}.png) and additional features - such as bathymetric contour lines - to help orientate and minimise problems in fishing ground identification, and (ii) printed in A4 paper size at 1:100,000 and 1:250,000 scales in order to provide interviewees with a choice.

Fishers, operating within the MPAs, were selected randomly through an opportunistic snowball sampling technique implemented during the on-field data collection campaigns. At the beginning of each interview, the interviewer explained the aims of the study, and respondents agreed to provide information for scientific purposes. Fishers were provided with the pre-print maps and they were asked to draw their own fishing grounds. Besides the identification of fishing grounds, interviewees were also required to quantify and characterise the use of the grounds in terms of specific fishing months, fishing days per month, employed fishing gear, fishing depth, and target species.

Overall, SSF data were collected from 71 out of the total 167 small-scale fishing vessels allowed to operate in the four Campanian MPAs, and samples were differently distributed among MPAs (Table 1). Interviews were carried out from October 2020 to July 2021, for a total of 22 days, asking information referring to the year 2019. The participatory mapping took approximately 15 min for each participating fisher. Therefore, 71 interviews were taken, i.e. one per fishing boat. They are not tied to a generic fishing trip but rather investigate what are the usual fishing grounds covered by each fisher. The fisher drew the area targeted by each of its fishing gears, and then explained how many fishing months he fished in 2019 with each gear. Additionally, the fisher specified the exact number of fishing days in each month.

**Table 1:** Composition of the sample for collecting SSF data (LOA < 12 m) in the MPAs case study area.

MPA	Fleet*	Sample**	Coverage***
Regno di Nettuno	73	38	52.1%
Punta Campanella	65	12	18.5%
Santa Maria di Castellabate	21	16	76.2%
Costa degli Infreschi e della Masseta	8	5	62.5%
**TOTAL**	**167**	**71**	**42.5%**

*n° of the total SSF vessels authorised to operate within each MPA.

**n° of SSF vessels whose fisher has been interviewed.

***sample/fleet*100.

The authors wish to emphasize that this study was a component of a broader research initiative, focused on examining the fishery sector of the Campania region. Therefore, in the supplementary material S1 only the questions relevant to this specific investigation have been included, together with an example of maps given to the fishers.

A web application was developed by using the Shiny R package^56^ to digitise drawings collected through face-to-face interviews and export digitised polygons as shapefiles. The application is accessible online at https://app.irbim.cnr.it/feampssfdatacollection/ssf_v0.0/ and default login credentials can be used (user: guest, password: guest23). It enabled any interviewer to quickly digitise their own sketches from the paper maps and store them in a common server, avoiding bias which may arise from the interpretation of the notes of others. Ongoing work aims to make the web app easier to use directly in the field (e.g., by using a tablet), hence supporting a completely digital data collection in the future.

**Fig. 3:**
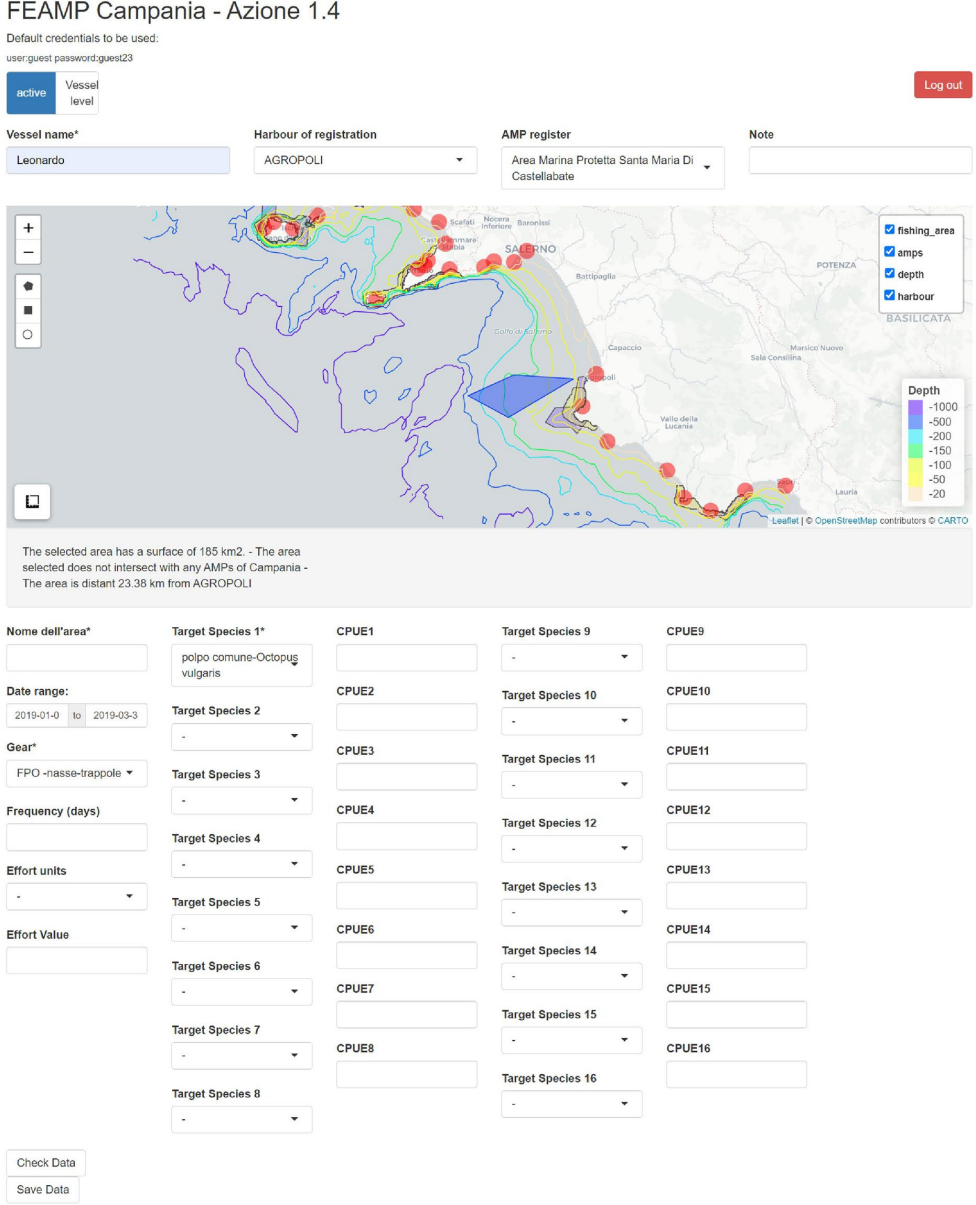
Developed web app interface. It allows drawing polygons, filling related attributes, and exporting as shapefiles.

The web app interface (Fig. 3) allows users to enable and disable different map layers (i.e., MPAs, contour lines and main ports) to orient themselves in digitising polygons, while custom text and multiple choice fields can be used to associate attribute information such as the name of the vessel, the port of registration, the registered MPA, the name of the area, the employed gear, the target species, the frequency (as the average number of fishing days per month), the fishing period and additional notes (comments or non-standardizable annotations). However, it was not always possible to fill out all the fields originally conceived in the interview or designed in the web application (e.g., the CPUE associated with each target species, the effort units, and the effort value), and thus such information was not reported in the final dataset.

The exported shapefile - containing all the SSF polygons and related attributes - was then processed in QGIS^57^ to aggregate effort/species/gear information to the same 0.01°x0.01° (1 km x 1 km) grid that was used to map the AIS-based estimated fishing effort. In particular, the grid was extended with additional gear-specific attributes on effort and targeted species by: (i) selecting by expression the employed gear from the shapefile containing all the polygons; (ii) creating temporary gear-specific grids with the number of intersecting polygons and the monthly and annual fishing days for each grid cell through the application of aggregation algorithms exploiting spatial criteria (e.g., *Join attributes by location, Join attributes by location (summary)*); (iii) selecting all the cells containing at least one polygon in each gear-specific grid; and (iv) joining by cell id the original 0.01°x0.01° (1 km x 1 km) grid and the temporary gear-specific grids, one at a time. Lastly, columns still referring to the original vessels and duplicated features were deleted, as well as grid cells intersecting MPAs’ zones A (as - due to their spatial resolution - these might result in fishing incorrectly allocated within banned zones). The final SSF grid layer stored information on effort and target species by gear type.

**Fig. 4:**
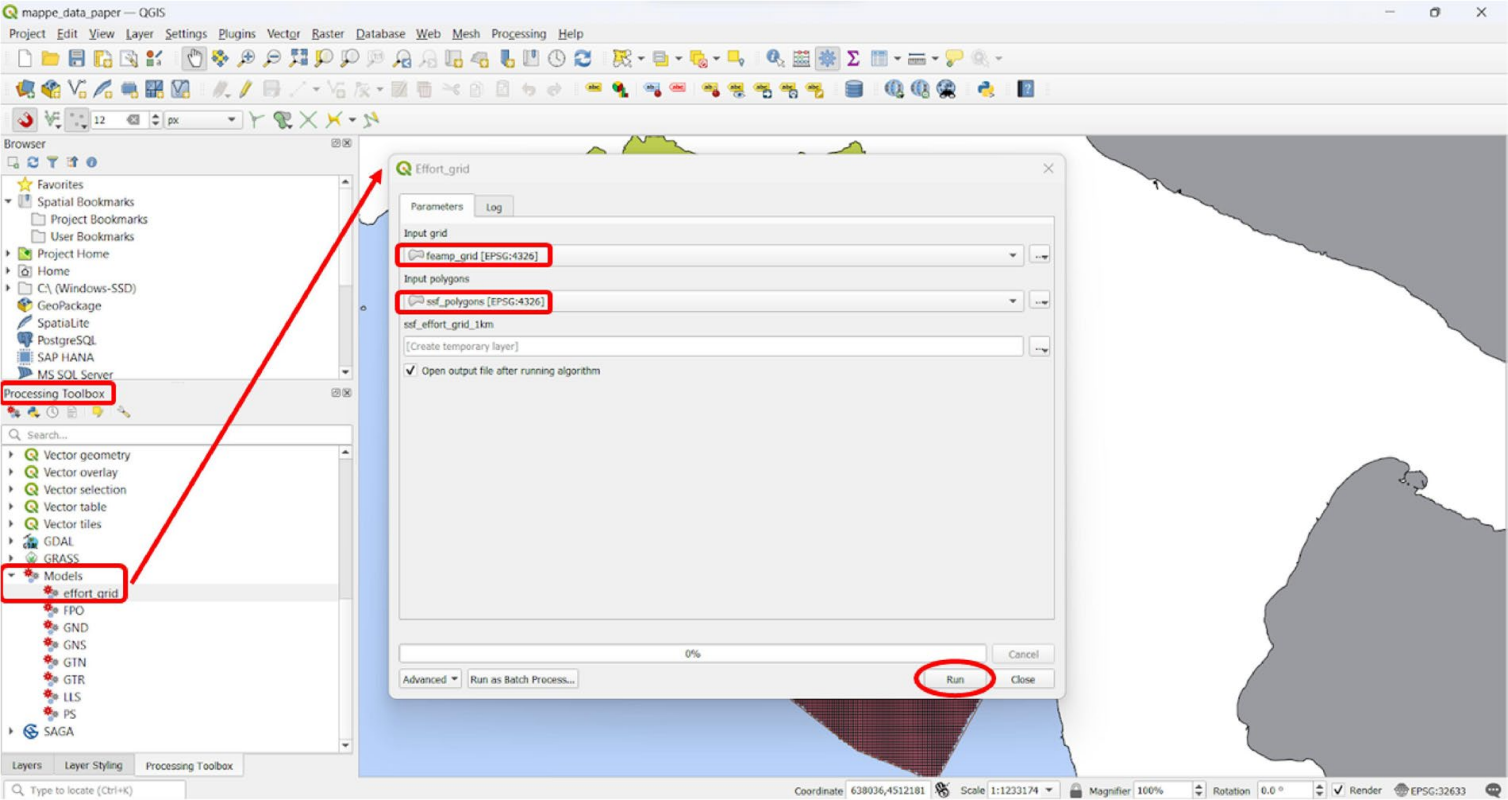
QGIS Graphical User Interface to run the released models, after having selected released data as input parameters (*Input grid* and *Input polygons*).

The chain of the abovementioned operations was wrapped into a single process using the QGIS graphical modeler (Fig. 4) and released at the same Figshare repository^46^ as .model3 file. It allows even novice GIS users to easily reproduce the workflow with their own set of inputs (i.e., digitised SSF polygons and aggregation grid), saving time and effort. A small guide is also shared (“step-by-step_guide.pdf”), to help users add the provided tools and run the final model “effort_grid.model3”.

## Data Records

The spatial dataset is accessible at the Figshare repository at 10.6084/m9.figshare.23592006 and accompanied by machine-readable metadata^46^. A summary table describing the variables of each dataset has been provided as Supplementary Table S2. The Italian version of the same is also available under request through the sharing services of the FEAMP GeoPortal (https://geoportal.bioinfo.szn.it/) and related GeoNetwork Catalogue, together with all the other outcomes of the project.

The dataset consists of a collection of five fishing effort files (Table 2): three shapefiles informing on LSF (N.1 at 0.05° × 0.05°–5 km × 5 km - resolution for the GSA10, and N.2 at 0.01° × 0.01°–1 km × 1 km - resolution within the MPAs case study area) and two shapefiles on SSF (consisting in the digitised polygons and the related gridded data at 0.01° × 0.01°–1 km × 1 km - within the MPAs case study area, respectively). In all the shapefiles, the attribute table displays the cell ID and the geographical coordinates of the vertices of each cell. The common lattice grid used within the case study area promotes the joining and overlaying of the released LSF and SSF effort data. A readme.txt file is provided at the figshare repository^46^, containing the table schemas and additional information.

**Table 2:** Table briefly summarising the features of each shared file. For additional information, see Supplementary Table S2.

Data product (shp format)	Fleet segment	Method	Spatial resolution*	Temporal resolution	Fishing effort indicator	Fishing gears
effort_grid_study_area_1km_month	LSF (LOA > 12 m) - 132 vessels involved	Tracking system - AIS data	0.01° × 0.01° (1 km × 1 km) - MPAs case study area	2019	Fishing hours, aggregated by month	OTB, PS
effort_grid_study_area_1km_month	LSF (LOA > 12 m) - 132 vessels involved	Tracking system - AIS data	0.01° × 0.01° (1 km × 1 km) - MPAs case study area	2019	Fishing hours, aggregated by year (even with the LOA categorization) + annual fishing hours quantified during AIS transmission gaps (hidden fishing activity)	OTB, PS
effort_grid_gsa10_5km	LSF (LOA > 12 m) - 334 vessels involved	Tracking system - AIS data	0.05° × 0.05° (5 km × 5 km) - GSA10	2019	Fishing hours, aggregated by year	OTB, PS
ssf_polygons	SSF (LOA < 12 m) - 71 vessels involved	Participatory mapping - interviews	—	2019	Fishing days, aggregated both by month and by year	Not-towed gears
ssf_effort_grid_1km	SSF (LOA < 12 m) - 71 vessels involved	Participatory mapping - interviews	0.01°×0.01° (1 km × 1 km) - MPAs case study area	2019	Fishing days, aggregated both by month and by year	Not-towed gears

*The spatial resolution has been adapted to the extension of the investigated area: 0.05° × 0.05° (5 km × 5 km) for the GSA10 vs. 0.01° × 0.01° (1 km × 1 km) for the MPAs case study area.

### Large-scale fisheries

As stated in Table 3, a total of 15177 fishing trips were documented by AIS in GSA10 in 2019, assigned to 334 fishing vessels and retrieved from the database. Focusing only on the MPAs case study area, 132 industrial fishing vessels were observed for a total of 6355 reconstructed fishing trips, of which 4185 were carried out by 53 Campanian vessels.

**Table 3:** Information from the selected AIS dataset in terms of number of vessels and fishing trips.

SELECTION	Fishing vessels	Fishing trips
Fleet GSA10	334	15177
Fleet FEAMP register	58*	5390
**Total**	**392**	**20567**
Fleet MPAs case study area (spatial overlay)	132 (53 from the FEAMP register)	6355 (4185 from the FEAMP register)

*Of the 58 FEAMP vessels analysed, one vessel was eliminated due to poor signal quality.

From the 1072 Campanian vessels listed in the FEAMP register, 62 vessels had an overall length above 15 m (LOA > = 15 m) and 60 of these ones were present within the AIS database (~97% of coverage).

In line with the fleet registers, most of the fishing trips were assigned to bottom otter trawlers (OTB), which predominated in the whole analysed fleet (~80%). Purse seine (PS) was the second most predicted gear (~15%), followed by the OTHER gear type (including gillnets, longlines, and dredges) that was considered negligible (and not released) (see Supplementary Tables S3, S4 and S5).

LSF fishing effort data in GSA10 was gridded at 0.05° × 0.05° (5 km × 5 km) and quantified in terms of (i) annual fishing hours that vessels spent operating each predicted gear (i.e., OTB or PS), and (ii) number of AIS broadcasts.

More detailed LSF fishing effort data were released in the study area, gridding at 0.01° × 0.01° (1 km × 1 km) and informing on (i) annual fishing hours that vessels spent operating each predicted gear (i.e., *OTB* or *PS*), (ii) annual fishing hours by fleet segment and predicted gear (e.g., *OTB_VL1218*: annual fishing hours that vessels between 12 m and 18 m in length spent operating trawl gears), (iii) monthly hours by predicted gears (e.g., *OTB_1*: fishing hours that trawlers spent in January operating trawl gear), (iv) annual unobserved fishing hours by predicted gear (e.g., *OTB_gaps*: annual trawling hours quantified during AIS transmission gaps), and (v) the number of AIS broadcasts.

In both grids, cells were further characterised by the overlay with the spatial restrictions envisaged by Council Regulation 1967/2006 (Mediterranean Regulation, MR) that prohibits trawl fishing within 3 nm of the coast or within the 50 m isobath (should that depth be reached at shorter length), and the purse seines within 300 m of the coast or within the 50 m isobath (should that depth be reached at shorter length). An additional overlay was carried out within the study area to distinguish grid cells within, intersecting and outside the MPAs. More details on these fields are given in the supplementarey Table S2 and in the readme.txt file stored in the figshare repository^46^.

Some examples of information that can be drawn for bottom trawlers by the released LSF data are shown hereafter.

**Fig. 5:**
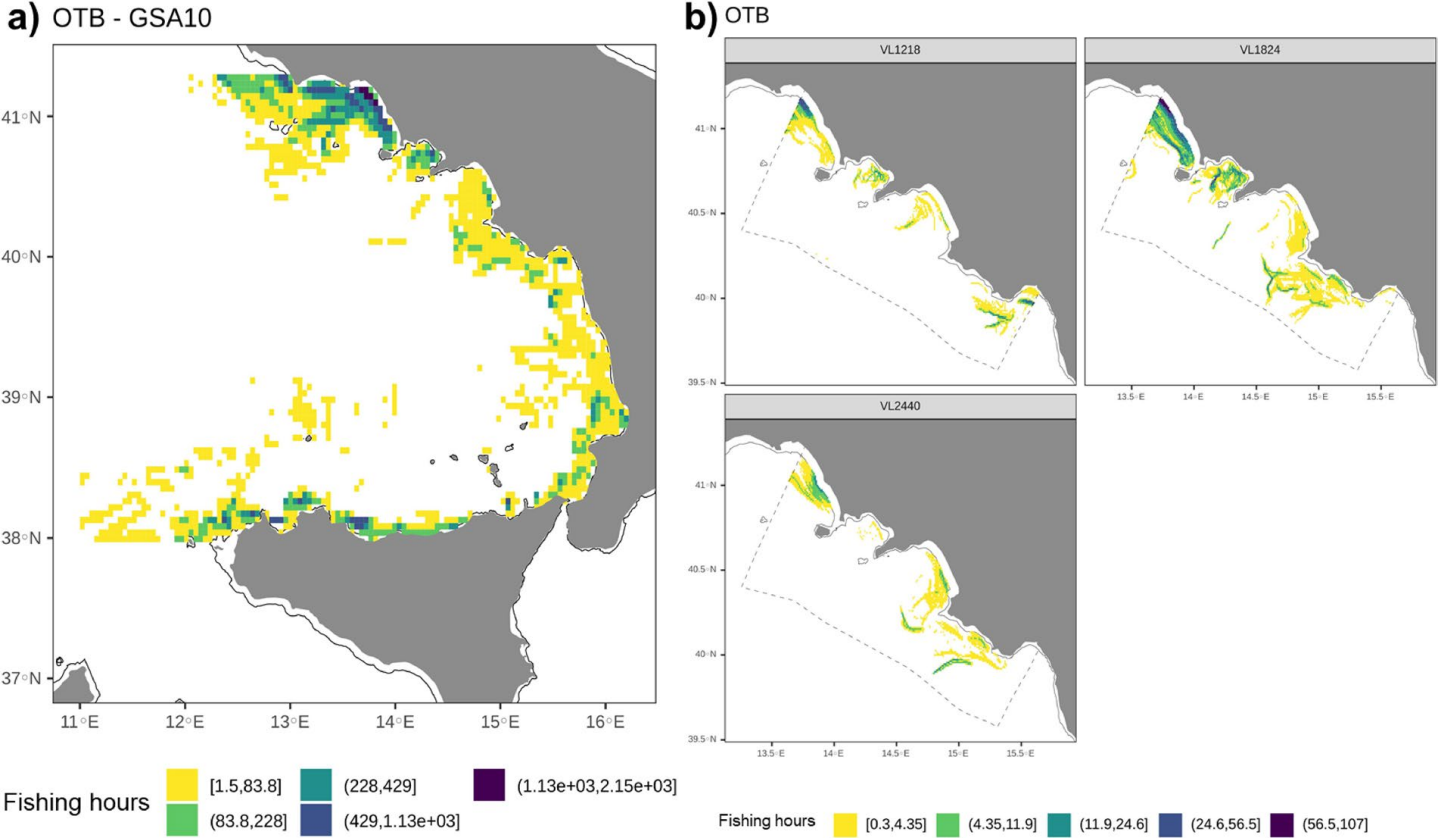
Annual fishing hours (year 2019) within GSA10 (left, grid resolution: 0.05°) and within the case study area by fleet segment (right, grid resolution: 0.01°).

From the annual pattern of fishing activity in GSA10 (in hours of fishing per square kilometres, Fig. 5a), we observed that most of the bottom otter trawling fishery was concentrated in the Gulf of Naples and off the north coast of Sicily, while other grounds are locally exploited in the Gulf of Gaeta. Investigating by vessel length (Fig. 5b), OTB fishing activity of the VL1218 segment was estimated in the whole study area, with a greater intensity in the northern and southern part (Gulf of Gaeta and Cilento) and a relatively lesser extent elsewhere (Gulf of Naples and Salerno). The fleet segment VL1824 was highly present in the whole study area, with the exception of the Gulf of Salerno where the trawling activity was relatively low. Bigger trawlers (VL2440) were instead observed in all the main fishing grounds of the study area with the exception of the Gulf of Naples, and to mainly exploit the deepest areas (e.g. Gulf of Salerno and Cilento).

**Fig. 6:**
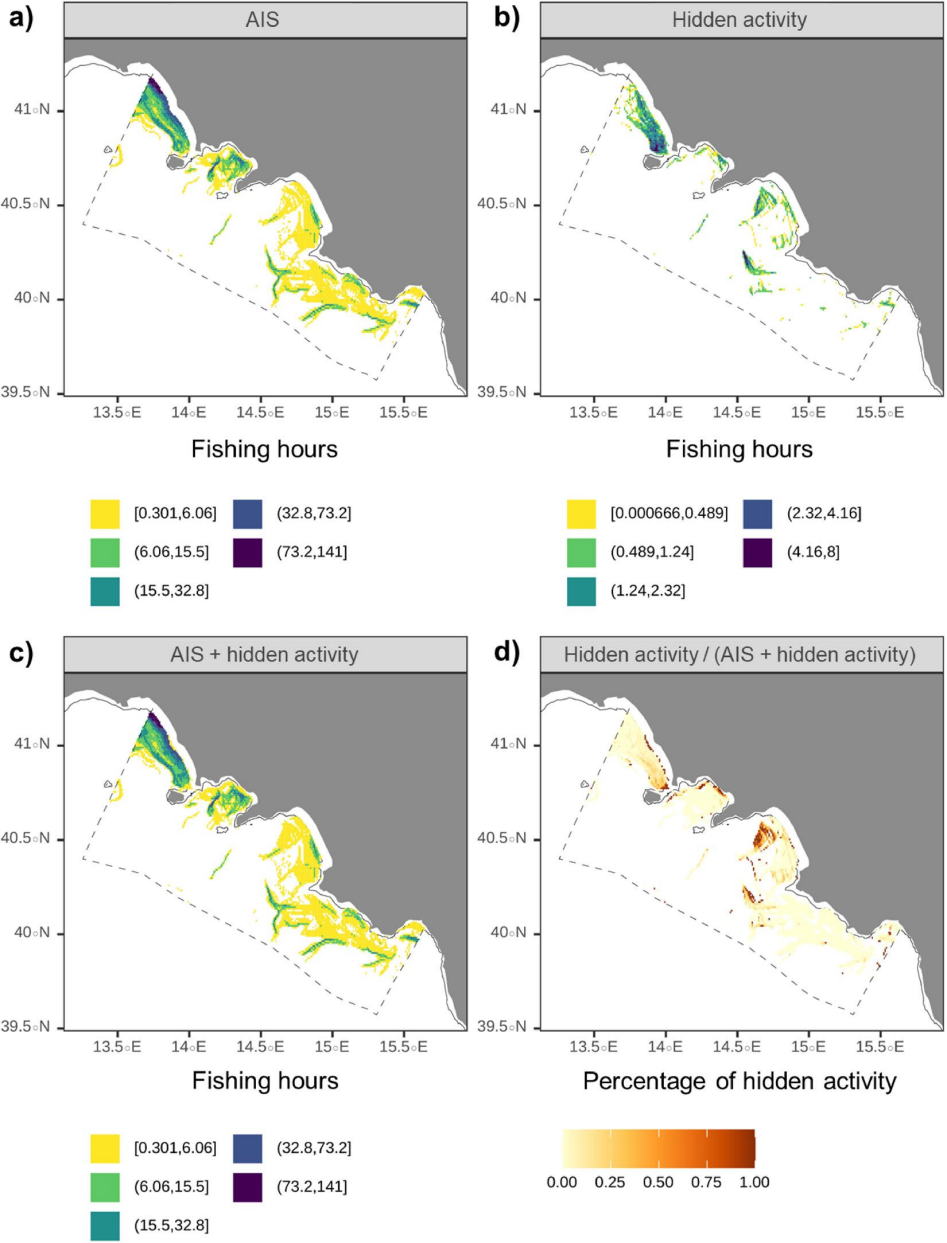
AIS-observed **(a)**, hidden potential **(b)** and total (observed + hidden) fishing activity **(c)** in the case study area (OTB, 2019), and related percentage of hidden fishing activity **(d)**.

Figure 6 depicts: (i) the trawling activity estimated from the observed AIS data (6a), (ii) the hidden activity compatible with the fishing (6b), (iii) the combination between the two (observed + hidden activity) (6c) and (iv) the percentage of hidden activity on the total (hidden activity / [observed + hidden activity]) (6d). The potential fishing subset added to the observer fishing increased the total fishing activity, showing some distinctive spatial patterns in the Gulf of Salerno, in the southern part of the Gulf of Gaeta and in the proximity of the 3 nm.

### Small-scale fisheries

**Fig. 7:**
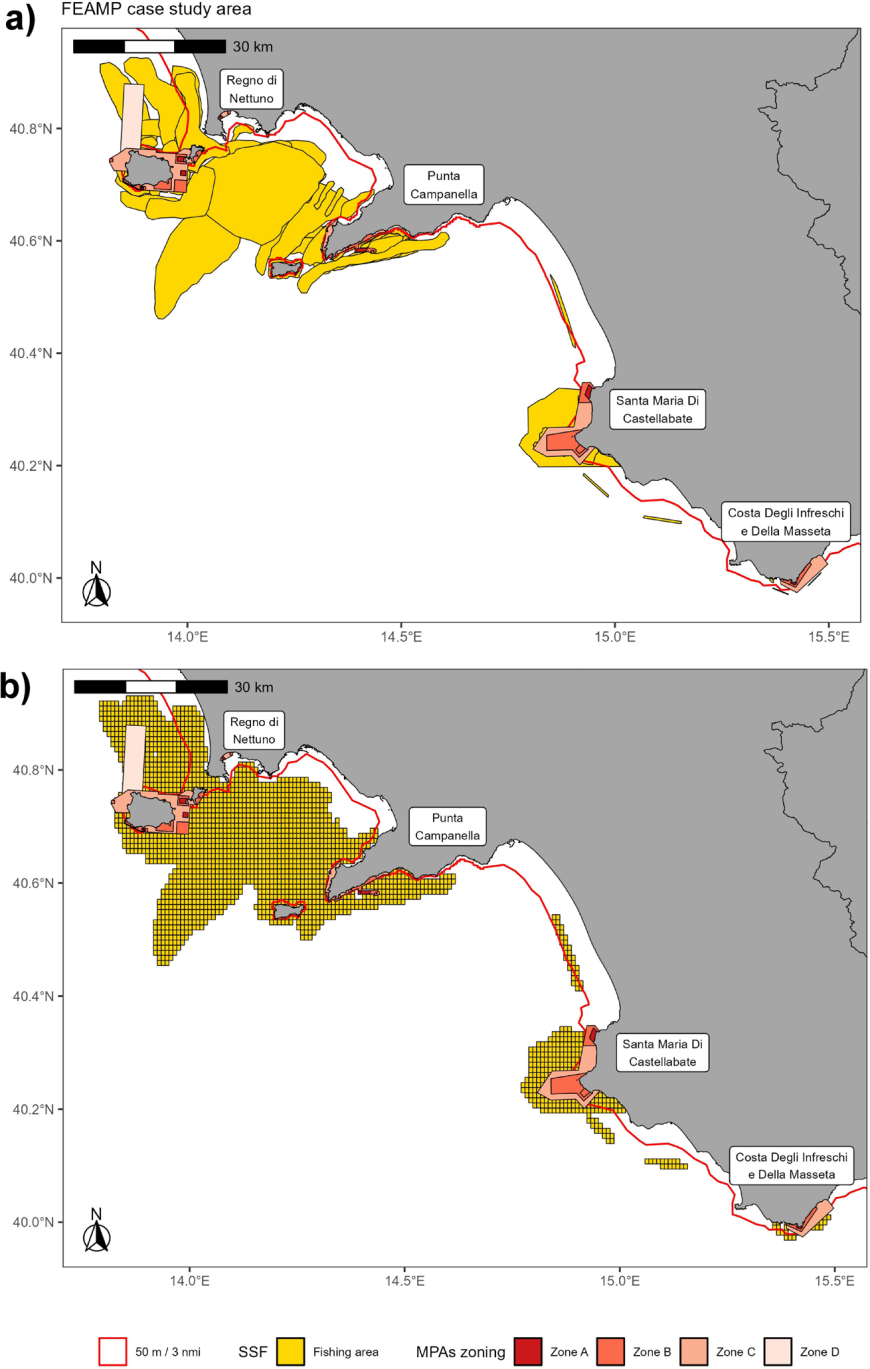
Digitized polygons **(a)** and aggregated SSF fishing effort (**b**, grid resolution: 0.01°). The trawl fishery ban (3nm from the coast or 50m isobath, where this is closer to the shoreline) is marked in red.

The 71 surveys - carried out between October 2020 and July 2021 - allowed to digitise 88 polygons (Fig. 7a) informing on the main fishing grounds, the employed gears and the targeted species. Each polygon was described with a unique gear, resulting in 29 vessels being associated with more than one polygon. Indeed, being SSF activities traditionally polyvalent and seasonally diverse, fishers replied to mostly use more than one gear and change gear according to the geographical areas and/or the period of the year.

SSF fishing effort data (Fig. 7b) were provided for the case study area (2328 0.01° × 0.01° grid cells, excluding cells overlapping MPA no-take/no-access zones), quantified in fishing days for each month and for the whole year related to each specific gear (e.g., *FPO_Jan, FPO_year*), and enriched in terms of: (i) the gears employed within each cell (*Gear*); (ii) the species fished within each cell, regardless of the gear used and provided both as Latin name and FAO 3-Alpha Species Code (*Species, Species_ab*)^58^; (iii) the MPAs where the contributing interviews were carried out (*MPA*) (e.g., *FPO_amp*, referring to a specific gear); (iv) the fished species for each employed gear, provided as both Latin name and FAO 3-Alpha Species Code (e.g., *FPO_sp, FPO_spabb*)^58^; (v) the number of vessels fishing in each cell with a specific gear (e.g., *FPO_count*), as they were derived from the overlapping polygons.

Seven distinct gears were recorded (pots/traps - FPO, purse seines - PS, gillnets - GNS, trammel nets - GTR, castellated/combined nets - GTN, driftnets - GND, and set longlines - LLS), with GNS the most represented (encountered in ~53% of the cells of the SSF effort grid), followed by LLS (~48%) and GTR (~26%).

A total of 51 target species were observed and registered in terms of: the Latin name and its shortened version, the English common name and the 3-Alpha Species Code nomenclature as proposed by FAO^58^ (Supplementary Table S6). The European hake (*Merluccius merluccius*) was fished in 79.7% of the grid cells of the SSF effort grid, followed by gurnards (*Chelidonichthys spp*., ~38.3%), common octopus (*Octopus vulgaris*, ~37.2%) and common cuttlefish (*Sepia officinalis*, ~27.4%).

**Fig. 8:**
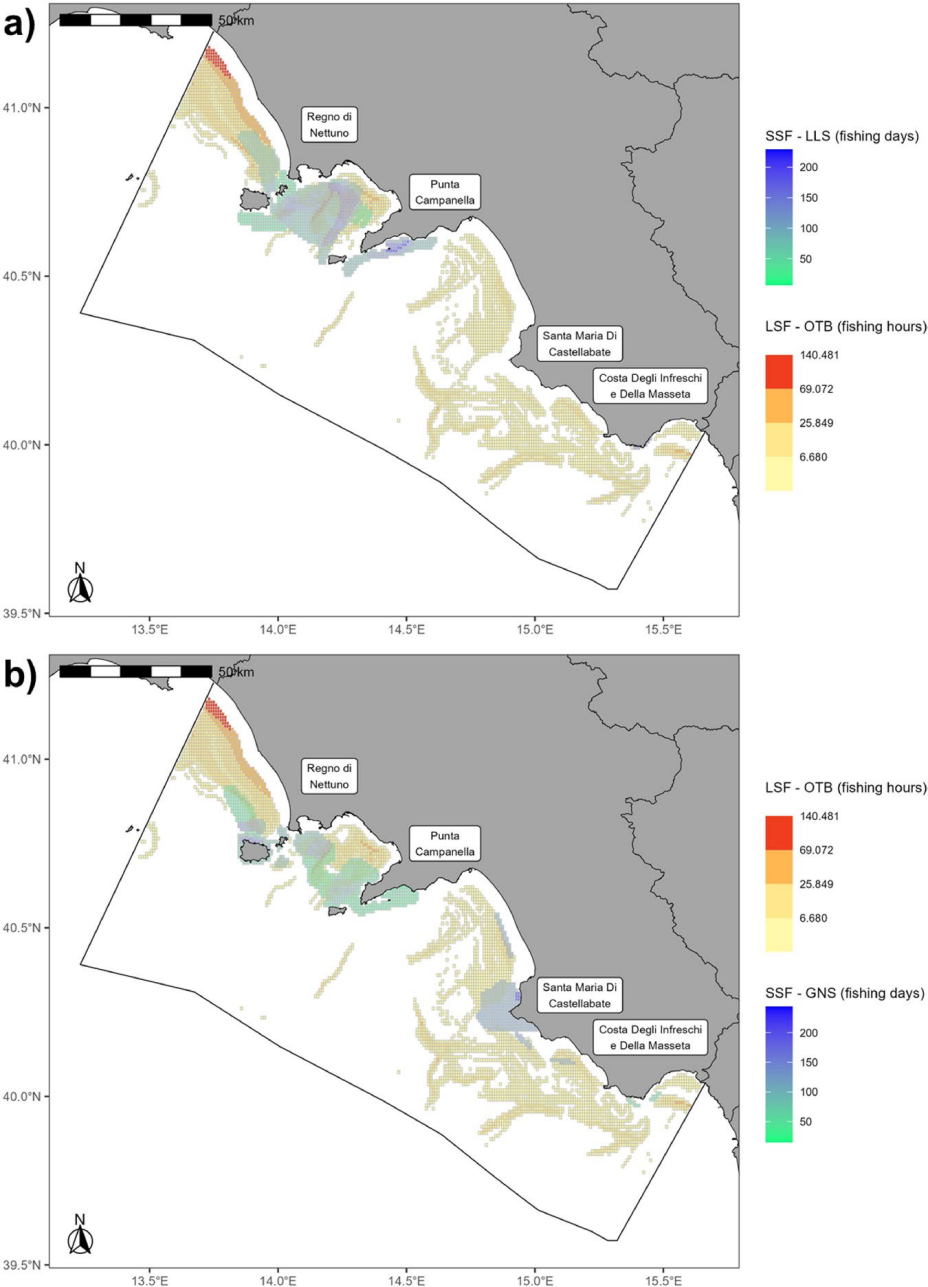
Spatial overlay between fishing activities carried by large-scale trawlers (LSF-OTB) and by small-scale LLS **(a)** and GNS **(b)**.

Most of the SSF fishing activity was exerted around Punta Campanella and Regno di Nettuno MPAs, and ~79% of the cells fell outside the 3 nm/50 m isobath trawling ban (Fig. 7), where the marine space and resources are shared with other fishing fleets, such as otter bottom trawlers (OTB). Figure 8 highlights spatial conflicts between SSFs and trawlers, especially around Punta Campanella and Regno di Nettuno MPAs).

**Fig. 9:**
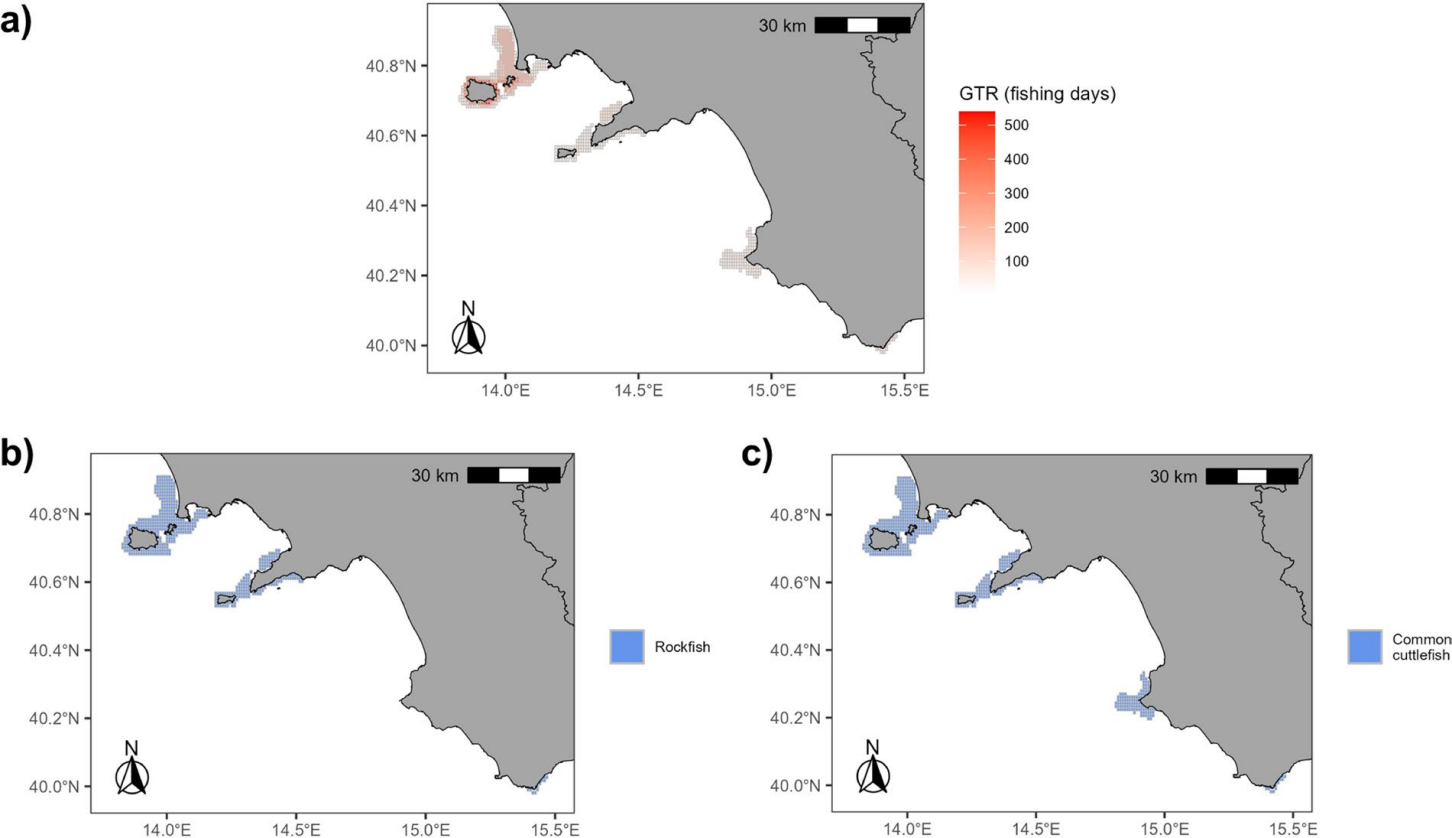
Spatial distribution of trammel nets activities **(a)** and related mostly targeted species **(b,c)**.

For some species, the correlation with the correspondent fishing gear is quite clear, and a few main examples are shown and detailed in Fig. 9 and Table 4 (i.e, rockfish and common cuttlefish with trammel nets, shortfin squid and monkfish with gillnets, and Mediterranean moray and European conger with pots/traps).

**Table 4:** Percentage of cells containing the most fished species by gears.

Gear	n. grid cells	Species	n. grid cells	Percentage
GTR	609	*Scorpaena spp*.	489	80.30%
GTR	609	*Sepia officinalis*	608	99.84%
GNS	1235	*Illex spp*.	455	36.84%
GNS	1235	*Lophius spp*.	257	20.81%
FPO	128	*Muraena helena*	128	100%
FPO	128	*Conger conger*	128	100%

## Technical Validation

The technical validation of LSF outcomes went through the matching procedure, which was necessary to associate AIS data with the technical-administrative information stored by the official fleet registers. Inferred fishing gears were in this way validated against vessel-specific licensed main and auxiliary gears. In the absence of logbooks and/or onboard observers, the information contained in these registers - net of the inaccuracies due to the lack of synchronisation between its updating and the vessel-specific events (e.g., change of name, codes, licence or place of registration) - represents indeed the only official source to validate AIS data processing and related inferred fishing patterns.

In particular, the classification performance was estimated according to a confusion matrix that reported the overlap between the AIS-estimated gear and the corresponding gear reported in the vessel’s registers. Considering the licensed gear as the ground truth, for each gear type *g* we then calculated: (i) *true positives* as the number of AIS-estimated *g* trips corresponding to official *g* types, (ii) *false positive*s as the number of AIS-estimated *g* trips not corresponding to official *g* types, (iii) *true negatives* as the number of AIS-estimated *non-g* trips that correspond to official *non-g* types; (iv) *false negatives* as the number of AIS-estimated *non-g* trips that correspond to official *g* types. Based on these metrics, classification performance was assessed in terms of: (i) *precision* = *true positives/(true positives* + *false positives), (ii) sensitivity* = *true positives/(true positives* + *false negatives), (iii) specificity* = *true negatives/(true negatives + false positives)*. Classification precisions ranged from 83% (OTHER) to 99% (OTB). A lower precision for PS (87%) was due to its confusion with OTB and OTHER categories. The classification algorithm showed the highest precision for OTB, which was also the category with the largest number of registered and estimated gear types. The high sensitivity for PS and OTB involved a generally small number of false negatives, except for OTHER (43% sensitivity), where the confusion was greater but uniform. The lower performance for OTHER was principally due to its under-representation in the training data. The generally high specificity across categories (96–98%) indicated a very high true negative detection, i.e. agreement on the fact that a trip did not use a given gear.

Finally, comparing LSF effort dataset with those made freely available by other sources (e.g., Global Fishing Watch^59^ and EMODnet MedSea Checkpoint^60^), similarities were noted underlining the consistency of the dataset released.

Regarding SSF data, and in accordance with the methodology used in earlier experiences^40^, validity was primarily evaluated on the spot by scientists or interviewers who gathered data on the same area from various fishermen and then double-checked it for potential discrepancies in terms of fishing gear, target species, and fishing days.

SSF digitised polygons were further validated by overlying the bathymetry and the substrate typology for each employed gear. Moreover, possible inconsistencies in collected spatially-explicit data were checked by experts of IREPA (Institute of Economic Research for Fisheries and Aquaculture), which also provided additional information for data-poor areas such as *Costa degli Infreschi e della Masseta*.

Based on this validation method, the authors are convinced that the suggested approach might be used in routine programs with little bias. Nonetheless, future investigations could attempt a quantitative evaluation of the reliability of the participatory maps in terms of agreement and consistency among experts^61,62^, thus improving the informational governance and quality standards.

## Usage Notes

All data and associated metadata are released under the Creative Commons Attribution licence (CC-BY, v. 4.0, https://creativecommons.org/licenses/by/4.0/deed.it, last access: 1 July 2023), and follow the FAIR principle of Findability, Accessibility, Interoperability and Reusability of data^63^. Data is available in .shp format and enriched with several attributes informing on gear, fishing segments and switching off (LSF dataset) and also on target species or metiers on a monthly/yearly timestamp (SSF dataset).

The dataset can be helpful to various end users, from policymakers to researchers, and different indicators of fishing effort have been provided so that the user who downloads the data can choose the indicator most suited to the study he/she is carrying out.

For instance, given the lack of accessible and spatially-explicit knowledge on small- compared with AIS-tracked large-scale fleets^64,65^, released data can spatially locate well-known conflicts between small- and large-scale fleets^40^ and provide fishery and MPA managers with baseline information for integrated coastal zone management and MSP, as well as with marine economic values-in-use for striking the ideal balance between conservation priorities and the requirements of the local population^48^.

From the resource management perspective, information on fished species could be valuable to understanding which stocks are exploited by both small-scale and large-scale fisheries. Moreover, it should be noteworthy that the selectivity and impacts of small-scale fishing gears are less studied - compared to other gears like trawls - as they are thought to be more selective and less damaging to stocks and habitats^66^.

Last, knowing where otter bottom trawlers operate could help in investigating potential impacts in ecologically relevant areas. This can be the case of coralligenous bioconstructions, as those located within the case study area^67^, that international protection agreements confirm as among the most endangered Mediterranean habitats (Habitat Directive 92/43/CEE; SPA/BIO Protocol; Barcelona Convention; Berne Convention). Their vulnerability is further exacerbated by their high levels of biodiversity, as they harbour a large variety of species including those highly sensitive to human disturbance^68^.

### Supplementary information

Supplementary Information

## Data Availability

The R code used to process AIS data was made available by previous Zenodo repositories^51,69^, while the QGIS model is deposited in the figshare public repository^46^ along with the released dataset. The R shiny web app is free of use using default login credentials (user: guest, password: guest23) and the related code will be released once it is finalised to be directly used in the field. All this could facilitate the adoption of the proposed integrated mapping approach and its tailoring in other contexts/case studies and decision-making frameworks.
