# Supplementary material for: Bridging the gap in fishing effort mapping: a spatially-explicit fisheries dataset for Campanian MPAs, Italy
Source: Sci Data. 2024 Jan 9;11:54. doi: 10.1038/s41597-023-02883-9 (PMC10776858; doi:10.1038/s41597-023-02883-9)
Supplement: Supplementary file 1 — Supplementary Information [file 41597_2023_2883_MOESM1_ESM.pdf]

Supplementary Information

Contents

Supplementary Material S1 2
Supplementary Table S2 10
Supplementary Table S3 31
Supplementary Table S4 32
Supplementary Table S5 33
Supplementary Table S6 34

Supplementary Material S1

The present document exemplifies the questions carried out and the sketch maps provided to the fishers during an interview in the Regno di Nettuno MPA.

SECTION 1 – INTERVIEWEE'S BASIC INFORMATION

Interview date	
Location	
Name of the vessel	
AMP where the vessel is registered	E.g., *Regno di Nettuno*
On average over the year, how many days a week do you fish inside and outside the marine protected area?	_____days fishing outside the MPA<br>_____days fishing inside the MPA

SECTION 2 – General information on the vessel

Vessel serial number				N° EU	
Tonnage		Power		Length	
Role of the interviewee				◯ Shipowner<br>◯ Commander<br>◯ Sailor / Cabin boy	
Does the owner also work on board the vessel?				◯ Yes<br>◯ No	
Number of crew members					
Base port					
Port of disembarkation					
Type of business				◯ Individual ◯ Cooperative (specify)	
Are you part of a consortium?				◯ No ◯ Yes (specify)	

SECTION 3 – Fishing activities and effort in 2019

Employed gear:	**GNS - gill nets**											
Year 2019	Jan	Feb	Mar	Apr	May	Jun	Jul	Aug	Sep	Oct	Nov	Dec
Number of fishing days within each month												
Caught species												
Bathymetry												
Fishing time (soak time)												
Mesh size												
Net length and height												
Filament type												
Notes												

Employed gear:	**GTR - trammel nets**											
Year 2019	Jan	Feb	Mar	Apr	May	Jun	Jul	Aug	Sep	Oct	Nov	Dec
Number of fishing days within each month												
Caught species												
Bathymetry												
Fishing time (soak time)												
Mesh size												
Net length and height												
Filament type												
Notes												

Employed gear:	**LLS - set longlines**											
Year 2019	Jan	Feb	Mar	Apr	May	Jun	Jul	Aug	Sep	Oct	Nov	Dec
Number of fishing days within each month												
Caught species												
Bathymetry												
Fishing time (soak time)												
Line length												
Distance of the arms												
Hooks dimension												
Notes												

Employed gear:	**FPO - pots and traps**											
Year 2019	Jan	Feb	Mar	Apr	May	Jun	Jul	Aug	Sep	Oct	Nov	Dec
Number of fishing days within each month												
Caught species												
Bathymetry												
Fishing time (soak time)												
Number of traps												
Mesh size												
Material												
Notes												

Employed gear:	PS - purse seines											
Year 2019	Jan	Feb	Mar	Apr	May	Jun	Jul	Aug	Sep	Oct	Nov	Dec
Number of fishing days within each month												
Caught species												
Bathymetry												
Fishing time (soak time)												
Notes												

Employed gear:	GTN - castellated/combined nets											
Year 2019	Jan	Feb	Mar	Apr	May	Jun	Jul	Aug	Sep	Oct	Nov	Dec
Number of fishing days within each month												
Caught species												
Bathymetry												
Fishing time (soak time)												
Notes												

Employed gear:	GND - driftnets											
Year 2019	Jan	Feb	Mar	Apr	May	Jun	Jul	Aug	Sep	Oct	Nov	Dec
Number of fishing days within each month												
Caught species												
Bathymetry												
Fishing time (soak time)												
Notes												

SECTION 4 – Spatial data

Which are the main exploited fishing areas?
Indicate the area drawing a polygon on one of the two provided maps, choosing the most appropriate zoom (1 polygon = 1 fishing gear used in a given period)
For each polygon, write down the following information:

- The polygon identifier (i.e., a number identifying the polygon as unique)
- The employed gear
- The vessel name
- Additional information (e.g., distance from the coast, name of the area)

(In the following pages maps illustrating the *Regno di Nettuno* MPA are shown as examples of maps provided during the interviews.)

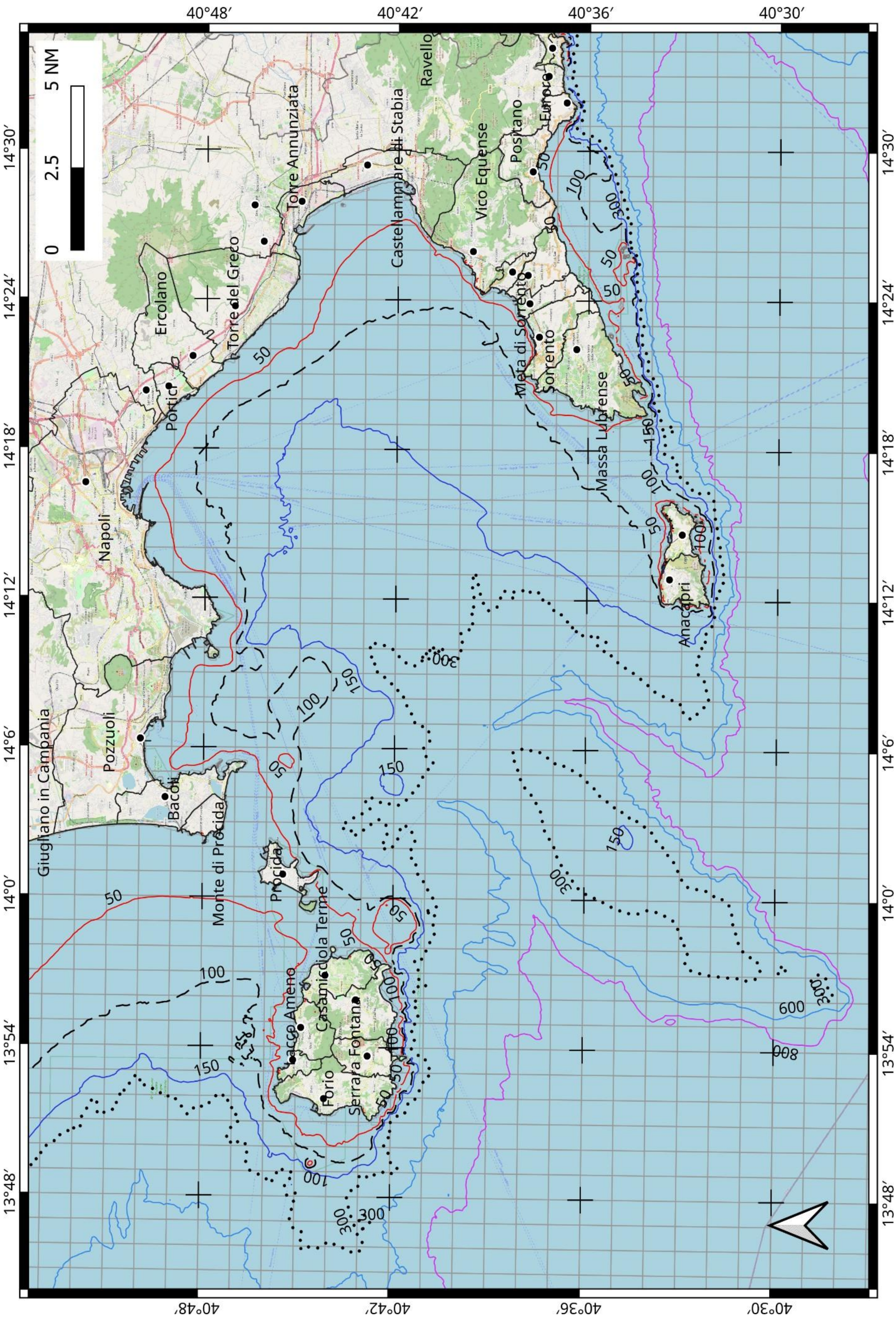
40°48′
40°42′
40°36′
40°30′
14°30′
14°24′
14°18′
14°12′
14°6′
14°0′
13°54′
13°48′
0
2.5
5 NM
Giugliano in Campania
Pozzuoli
Bacoli
Monte di Procida
Procida
Napoli
Portici
Ercolano
Torre del Greco
Torre Annunziata
Castellammare di Stabia
Ravello
Vico Equense
Positano
Furore
Meta di Sorrento
Sorrento
Massa Lubrense
Anacapri
Casamicciola Terme
Lacco Ameno
Forio
Serrara Fontana

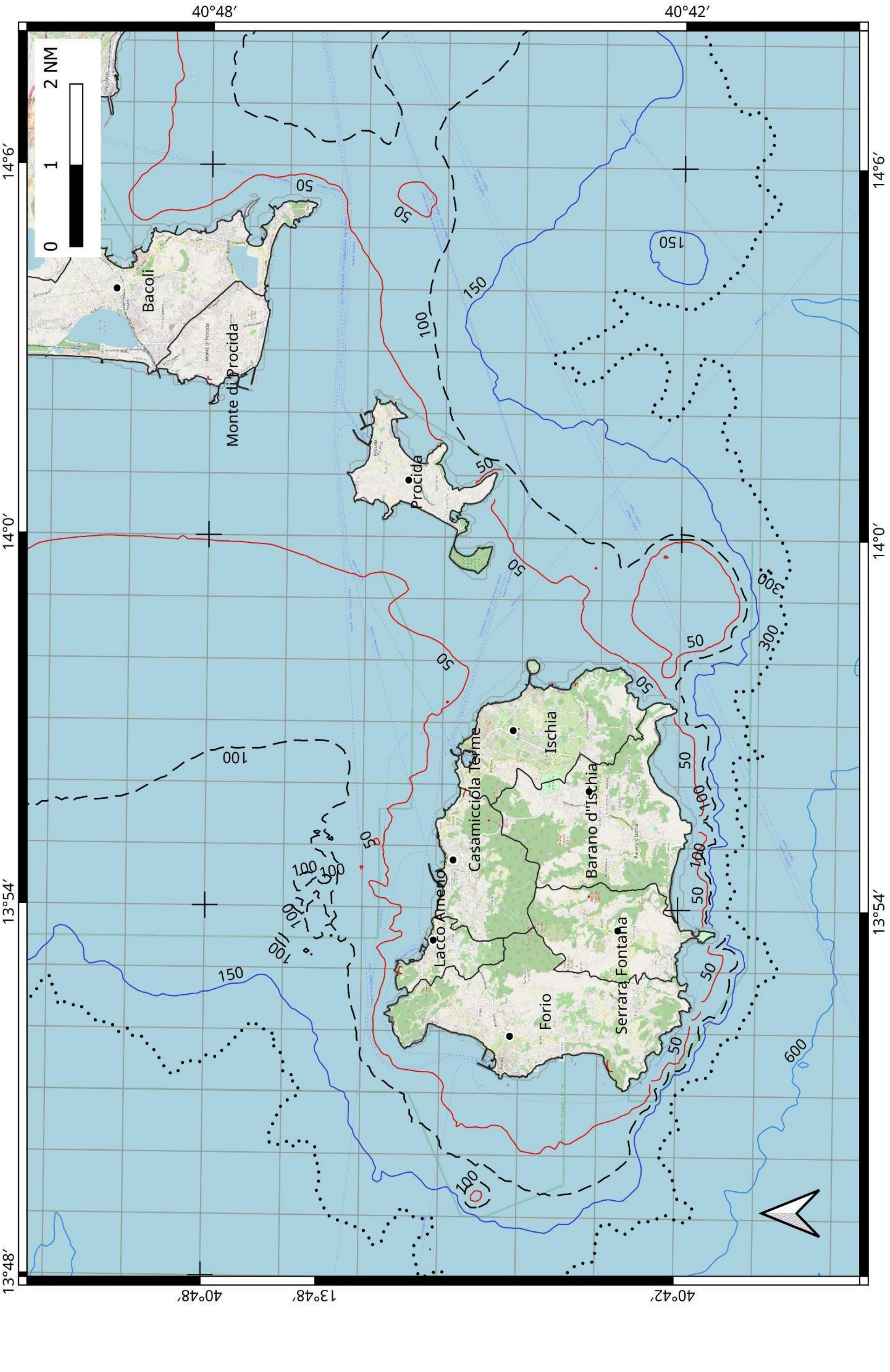

40°48′
40°42′
14°6′
14°0′
13°54′
13°48′
0
1
2 NM
Bacoli
Monte di Procida
Procida
Ischia
Casamicciola Terme
Barano d'Ischia
Lacco Ameno
Forio
Serrara Fontana
50
100
150
300
600

Supplementary Table S2

Table briefly explaining features of the shared data products available at the figshare repository.

n.	Dataset	File name	Brief description	Format	Licence	Temporal range of observations	Processing methodology	Number of records	Bounding box	Sea Area Common Name	CRS	Parameters
1	Case study grid - 0.01°x0.01° grid resolution	feamp_grid.shp	0.01°x0.01° grid of the case study area. It was used to aggregate data.	ESRI Shapefile	Creative Commons Attribution licence (CC-BY, v. 4.0, https://creativecommons.org/licenses/by/4.0/deed.it, last access: 3 May 2023)	2019	The polygon grid was created using the QGIS Create Grid tool (under Vector > Research Tools > Create Grid)	33809	N, S, E, W = 41.299999462, 39.572137702, 15.814614878, 13.249820078 [decimal deg]	Case study area within GSA 10-Southern and Central Tyrrhenian Sea	WGS84	*id: grid cell identification code<br>*xmin: longitude of the upper left vertex of the cell<br>*xmax: longitude of the lower right vertex of the cell<br>*ymin: latitude of the lower right vertex of the cell<br>*ymax: latitude of the upper left vertex of the cell
2	Monthly AIS density and estimated fishing effort in the study area - 0.01°x0.01° grid resolution	effort_grid_study_area_1km_month.shp	Monthly density of AIS signal (number of pings) and estimated fishing activities (in fishing hours) of bottom otter trawl (OTB) and purse seine (PS) from AIS 2019 in the study area - 0.01°x0.01° grid resolution. All grid cells	ESRI Shapefile	Creative Commons Attribution licence (CC-BY, v. 4.0, https://creativecommons.org/licenses/by/4.0/deed.it, last access: 3 May 2023)	2019	Analysis was based on the terrestrial AIS (year 2019), owned by CNR-IRBIM and obtained from a private provider (http://www.astrapaging.com/), with a poll frequency of 5 min and including EU and non-EU vessels (AIS type = 30).The R	33809	N, S, E, W = 41.299999462, 39.572137702, 15.814614878, 13.249820078 [decimal deg]	Case study area within GSA 10-Southern and Central Tyrrhenian Sea	WGS84	*id: grid cell identification code (same values of "grid_id" field)<br>*xmin: longitude of the upper left vertex of the cell<br>*xmax: longitude of the lower right vertex of the cell<br>*ymin: latitude of the lower right vertex of the cell<br>*ymax: latitude of the upper left vertex of the cell<br>*amp: 0 = cells outside the MPA, 1 = cells within the MPA, 2 = cells intersecting the MPA boundaries

			with estimated fishing effort less than 0.3 fishing hours were excluded.				code used to process AIS data is available at: http://doi.org/10.5281/zenodo.4761890					*manag_OTB: 0 = cells outside the 3 nm of the coast or the 50 m isobath (should that depth be reached at shorter length), 1 = cells within the 3 nm/50 m isobath, 2 = cells intersecting the 3 nm/50 m isobath

n.	Dataset	File name	Brief description	Format	Licence	Temporal range of observations	Processing methodology	Number of records	Bounding box	Sea Area Common Name	CRS	Parameters
												*manag_PS: 1 = cells within within the 300m of the coast or the 50 m isobath (should that depth be reached at shorter length); 2 = cells interesting the 300m/50 m isobath<br>*OTB_1: fishing hours related to January for bottom otter trawls<br>*OTB_2: fishing hours related to February for bottom otter trawls<br>*OTB_3: fishing hours related to March for bottom otter trawls<br>*OTB_4: fishing hours related to April for bottom otter trawls<br>*OTB_5: fishing hours related to May for bottom otter trawls<br>*OTB_6: fishing hours related to June for bottom otter trawls<br>*OTB_7: fishing hours related to July for bottom otter trawls<br>*OTB_8: fishing hours related to August for bottom otter trawls<br>*OTB_9: fishing hours related to September for bottom otter trawls<br>*OTB_10: fishing hours related to October for bottom otter trawls<br>*OTB_11: fishing hours related to November for bottom otter trawls

n.	Dataset	File name	Brief description	Format	Licence	Temporal range of observations	Processing methodology	Number of records	Bounding box	Sea Area Common Name	CRS	Parameters
												*OTB_12: fishing hours related to December for bottom otter trawls<br><br>*PS_1: fishing hours related to January for purse seines<br><br>*PS_2: fishing hours related to February for purse seines<br><br>*PS_3: fishing hours related to March for purse seines<br><br>*PS_4: fishing hours related to April for purse seines<br><br>*PS_5: fishing hours related to May for purse seines<br><br>*PS_6: fishing hours related to June for purse seines<br><br>*PS_7: fishing hours related to July for purse seines<br><br>*PS_8: fishing hours related to August for purse seines<br>*PS_9: fishing hours related to September for purse seines<br>*PS_10: fishing hours related to October for purse seines<br>*PS_11: fishing hours related to November for purse seines<br>*PS_12: fishing hours related to December for purse seines

n.	Dataset	File name	Brief description	Format	Licence	Temporal range of observations	Processing methodology	Number of records	Bounding box	Sea Area Common Name	CRS	Parameters
3	AIS density and estimated fishing effort in the study area - 0.01°x0.01° grid resolution	effort_grid_study_area_1km.shp	Density of AIS signal (number of pings) and estimated fishing activities (in fishing hours) of bottom otter trawl (OTB) and purse seine (PS) from AIS data 2019 in the study area. The estimation of fishing effort includes also fishing activity estimated by gaps analysis and by fleet segments (VESSEL_LENGTH classes) - 0.01°x0.01° grid resolution. All grid cells with estimated fishing effort less than 0.3 hours were excluded.	ESRI Shapefile	Creative Commons Attribution licence (CC-BY, v. 4.0, https://creativecommons.org/licenses/by/4.0/deed.it, last access: 3 May 2023)	2019	Analysis was based on the terrestrial AIS (year 2019), owned by CNR-IRBIM and obtained from a private provider (http://www.astrapaging.com/), with a poll frequency of 5 min and including EU and non-EU vessels (AIS type = 30).The R code used to process AIS data is available at: http://doi.org/10.5281/zenodo.4761890	33809	N, S, E, W = 41.299999462, 39.572137702, 15.814614878, 13.249820078 [decimal deg]	Case study area within GSA 10- Southern and Central Tyrrhenian Sea	WGS84	*id: grid cell identification code (same values of “grid_id” field)<br>*xmin: longitude of the upper left vertex of the cell<br>*xmax: longitude of the lower right vertex of the cell<br>*ymin: latitude of the lower right vertex of the cell<br>*ymax: latitude of the upper left vertex of the cell<br>*amp: 0 = cells outside the MPAs, 1 = cells within the MPAs, 2 = cells along the edge of the MPAs<br>*manag_OTB: 0 = cells outside the 3 nm of the coast or the 50 m isobath (should that depth be reached at shorter length), 1 = cells within the 3 nm/50 m isobath, 2 = cells intersecting the 3 nm/50 m isobath<br>*manag_PS: 1 = cells within within the 300m of the coast or the 50 m isobath (should that depth be reached at shorter length); 2 = cells interesting the 300m/50 m isobath<br>*OTB: annual fishing hours exerted by bottom otter trawlers for that cell<br>*PS: annual fishing hours exerted by purse seiners for that cell

n.	Dataset	File name	Brief description	Format	Licence	Temporal range of observations	Processing methodology	Number of records	Bounding box	Sea Area Common Name	CRS	Parameters
												*OTB_VL1218: annual fishing hours exerted by bottom otter trawlers between 12 m and 18 m in length<br>*OTB_VL1824: annual fishing hours exerted by bottom otter trawlers between 18 m and 24 m in length<br>*OTB_VL2440: annual fishing hours exerted by bottom otter trawlers between 24 m and 40 m in length<br>*PS_VL1218: annual fishing hours exerted by purse seiners between 12 m and 18 m in length<br>*PS_VL1824: annual fishing hours exerted by purse seiners between 18 m and 24 m in length<br>*PS_VL2440: annual fishing hours exerted by purse seiners between 24 m and 40 m in length<br>*PS_VL40XX: annual fishing hours exerted by purse seiners greater than 40 m in length<br>*OTB_gaps: annual fishing hours quantified for trawlers during AIS transmission gaps (hidden fishing activities)<br>*PS_gaps: annual fishing hours quantified for purse seiners during AIS transmission gaps (hidden fishing activities)

n.	Dataset	File name	Brief description	Format	Licence	Temporal range of observations	Processing methodology	Number of records	Bounding box	Sea Area Common Name	CRS	Parameters
												*density: the logarithm of the AIS broadcasts for each single cell
4	AIS density and estimated fishing effort in GSA 10 - 0.05°x0.05° grid resolution	effort_grid_gsa10_5km.shp	Density of AIS signal (number of pings) and estimated fishing activities (in fishing hours) of bottom otter trawl (OTB) and purse seine (PS) from AIS 2019 in GSA 10 - 0.05°x0.05° grid resolution. All grid cells with estimated fishing effort less than 0.5 were excluded.	ESRI Shapefile	Creative Commons Attribution licence (CC-BY, v. 4.0, https://creativecommons.org/licenses/by/4.0/deed.it, last access: 3 May 2023)	2019	AIS data 2019 were bought from a private provider and processed according to the R code available at: http://doi.org/10.5281/zenodo.4761890	7099	N, S, E, W = 41.299999462, 37.970265862, 16.264578878, 11.000000078 [decimal deg]	GSA 10 - Southern and Central Tyrrhenian Sea	WGS84	*id: grid cell identification code (same values of "grid_id" field)<br>*xmin: longitude of the upper left vertex of the cell<br><br>*xmax: longitude of the lower right vertex of the cell<br><br>*ymin: latitude of the lower right vertex of the cell<br>*ymax: latitude of the upper left vertex of the cell<br><br>*manag: 1= cells inside the 3 nautical miles (50 m depth) limit, 2 = cells overlapping with the 3 nautical miles (50 m depth) limit<br>*OTB: annual fishing hours exerted by bottom otter trawls for that cell<br>*PS: annual fishing hours exerted by purse seiners for that cell<br><br>*density: the logarithm of the AIS broadcasts for that cell
5	Participatory mapping of small scale fisheries - polygons	ssf_polygons.shp	Participatory mapping of small-scale fishing. Small-scale fishing effort is provided in the form of polygons, as they were	ESRI Shapefile	Creative Commons Attribution licence (CC-BY, v. 4.0, https://creativecommons.org/licenses/by/4.0/deed.it, last	2019	A participatory mapping approach was chosen for characterizing the SSF segment and mapping its fishing grounds in the proximity of the 4 MPAs	88	N, S, E, W = 40.926092475, 39.970411940, 15.482430105, 13.795764076 [decimal deg]	Case study area within GSA 10-Southern and Central Tyrrhenian Sea	WGS84	* id: identification code of the polygon (unique)<br>*id_sbpl: number assigned to each polygon within one of the four study areas (the number is the same for polygons with the same geometry)<br>* dt_strt: start date of fishing with that gear

n.	Dataset	File name	Brief description	Format	Licence	Temporal range of observations	Processing methodology	Number of records	Bounding box	Sea Area Common Name	CRS	Parameters
			drawn by fishermen during the interviews. The dataset contains information about the fishing effort in terms of fishing days (per month and per year), related gear, and targeted species.		access: 3 May 2023)		of GFCM GSA 10: Regno di Nettuno, Punta Campanella, Santa Maria di Castellabate, and Costa degli Infreschi e della Masseta. Data was collected through individual semi-structured face-to-face interviews with 71 fishers (out of 167 small-fishing vessels authorized to fish within the 4 MPAs) and then digitized using the develop web app.					* date_nd: end date of fishing with that gear<br>* gear: employed gear in that specific polygon<br>* vessel: name of the vessel associated to that specific polygon<br>* hrbr_rg: port where the vessel is registered<br>* amps_rg: MPA within which the vessel is fishing<br>* dt_cllc: date of data entry by the user<br>* usr_cll: user who is entering the data<br>*list_sp: list of species (common names) fished in that polygon through that gear<br>*list_sp_ab: list of species (3-Alpha Species Codes - according to the nomenclature proposed by FAO) fished in that polygon through that gear<br>*Jan_d: fishing days for the month of January referring to that polygon<br>*Feb_d: fishing days for the month of February referring to that polygon<br>*Mar_d: fishing days for the month of March referring to that polygon<br>*Apr_d: fishing days for the month of April referring to that polygon<br>*May_d: fishing days for the month of May referring to that polygon

n.	Dataset	File name	Brief description	Format	Licence	Temporal range of observations	Processing methodology	Number of records	Bounding box	Sea Area Common Name	CRS	Parameters
												*Jun_d: fishing days for the month of June referring to that polygon<br>*Jul_d: fishing days for the month of July referring to that polygon<br>*Aug_d: fishing days for the month of August referring to that polygon<br>*Sep_d: fishing days for the month of September referring to that polygon<br>*Oct_d: fishing days for the month of October referring to that polygon<br>*Nov_d: fishing days for the month of November referring to that polygon<br>*Dec_d: fishing days for the month of December referring to that polygon<br>*Days_year: annual fishing days referring to that polygon (sum of the fishing days from the various months)
6	Participatory mapping of small scale fisheries - 0.01°x0.01° grid resolution	ssf_effort_grid_1km.shp	Participatory mapping of small-scale fishing. Small-scale fishing effort is provided with a resolution of 0.01°*0.01°, in terms of fishing days, related employed gear, and targeted species.	ESRI Shapefile	Creative Commons Attribution licence (CC-BY, v. 4.0, https://creativecommons.org/licenses/by/4.0/deed.it, last access: 3 May 2023)	2019	A participatory approach was chosen for characterizing the SSF segment and mapping its fishing grounds in the proximity of the 4 MPAs of GFCM GSA 10: Regno di Nettuno, Punta Campanella, Santa Maria di Castellabate, and	2328	N, S, E, W = 40.931028982, 39.968106022, 15.490640798, 13.789776878 [decimal deg]	Case study area within GSA 10-Southern and Central Tyrrhenian Sea	WGS84	*id_cell: grid cell identification code<br>*xmin: longitude of the upper left vertex of the cell<br><br>*xmax: longitude of the lower right vertex of the cell<br><br>*ymin: latitude of the lower right vertex of the cell<br>*ymax: latitude of the upper left vertex of the cell<br>*FPO_Jan: fishing days related to January for pots/traps

n.	Dataset	File name	Brief description	Format	Licence	Temporal range of observations	Processing methodology	Number of records	Bounding box	Sea Area Common Name	CRS	Parameters
							Costa degli Infreschi e della Masseta. Polygons, collected through individual semi-structured face-to-face interviews with 71 fishers (out of 167 small-fishing vessels authorized to fish within the 4 MPAs), were processed in QGIS developing ad hoc models, aggregating on a standardized 0.01°x0.01° grid and evaluating the fishing effort (in fishing days) based on the employed gear.					*FPO_Feb: fishing days related to February for pots/traps<br>*FPO_Mar: fishing days related to March for pots/traps<br>*FPO_Apr: fishing days related to April for pots/traps<br>*FPO_Mag: fishing days related to May for pots/traps<br>*FPO_Jun: fishing days related to June for pots/traps<br>*FPO_Jul: fishing days related to July for pots/traps<br>*FPO_Aug: fishing days related to August for pots/traps<br>*FPO_Sep: fishing days related to September for pots/traps<br>*FPO_Oct: fishing days related to October for pots/traps<br>*FPO_Nov: fishing days related to November for pots/traps<br>*FPO_Dec: fishing days related to December for pots/traps<br>*FPO_year: annual fishing days resulting from the sum of the monthly fishing days for pots/traps<br>*FPO_count: sum of the vessels (polygons) insisting on that cell for pots/traps

n.	Dataset	File name	Brief description	Format	Licence	Temporal range of observations	Processing methodology	Number of records	Bounding box	Sea Area Common Name	CRS	Parameters
												*FPO_amp: belonging area; it depends on the original polygons employing pots/traps, and, therefore, on where the interview was made (indeed, some cells may be located between two areas)<br><br>*FPO_spabb: species fished in that cell through pots/traps - abbreviated species names following the FAO nomenclature based on 3-Alpha Species Codes<br>*FPO_sp: species fished in that cell through pots/traps<br><br>*PS_Jan: fishing days related to January for purse seines<br>*PS_Feb: fishing days related to February for purse seines<br>*PS_Mar: fishing days related to March for purse seines<br>*PS_Apr: fishing days related to April for purse seines<br>*PS_Mag: fishing days related to May for purse seines<br>*PS_Jun: fishing days related to June for purse seines<br><br>*PS_Jul: fishing days related to July for purse seines<br>*PS_Aug: fishing days related to August for purse seines

n.	Dataset	File name	Brief description	Format	Licence	Temporal range of observations	Processing methodology	Number of records	Bounding box	Sea Area Common Name	CRS	Parameters
												*PS_Sep: fishing days related to September for purse seines<br>*PS_Oct: fishing days related to October for purse seines<br>*PS_Nov: fishing days related to November for purse seines<br>*PS_Dec: fishing days related to December for purse seines<br>*PS_year: annual fishing days resulting from the sum of the monthly fishing days for purse seines<br>*PS_count: sum of the vessels (polygons) insisting on that cell for purse seines<br>*PS_amp: belonging area; it depends on the original polygons employing purse seines, and, therefore, on where the interview was made (indeed, some cells may be located between two areas)<br><br>*PS_spabb: species fished in that cell through purse seines - abbreviated species names following the FAO nomenclature based on 3-Alpha Species Codes<br><br>*PS_sp: species fished in that cell through purse seines<br><br>*GNS_Jan: fishing days related to January for gillnets

n.	Dataset	File name	Brief description	Format	Licence	Temporal range of observations	Processing methodology	Number of records	Bounding box	Sea Area Common Name	CRS	Parameters
												*GNS_Feb: fishing days related to February for gillnets<br><br>*GNS_Mar: fishing days related to March for gillnets<br>*GNS_Apr: fishing days related to April for gillnets<br>*GNS_Mag: fishing days related to May for gillnets<br>*GNS_Jun: fishing days related to June for gillnets<br>*GNS_Jul: fishing days related to July for gillnets<br><br>*GNS_Aug: fishing days related to August for gillnets<br>*GNS_Sep: fishing days related to September for gillnets<br><br>*GNS_Oct: fishing days related to October for gillnets<br>*GNS_Nov: fishing days related to November for gillnets<br>*GNS_Dec: fishing days related to December for gillnets<br>*GNS_year: annual fishing days resulting from the sum of the monthly fishing days for gillnets<br>*GNS_count: sum of the vessels (polygons) insisting on that cell for gillnets

n.	Dataset	File name	Brief description	Format	Licence	Temporal range of observations	Processing methodology	Number of records	Bounding box	Sea Area Common Name	CRS	Parameters
												*GNS_amp: belonging area; it depends on the original polygons employing gillnets, and, therefore, on where the interview was made (indeed, some cells may be located between two areas)<br><br>*GNS_spabb: species fished in that cell through gillnets - abbreviated species names following the FAO nomenclature based on 3-Alpha Species Codes<br>*GNS_sp: species fished in that cell through gillnets<br>*GTR_Jan: fishing days related to January for trammel nets<br>*GTR_Feb: fishing days related to February for trammel nets<br>*GTR_Mar: fishing days related to March for trammel nets<br>*GTR_Apr: fishing days related to April for trammel nets<br>*GTR_Mag: fishing days related to May for trammel nets<br>*GTR_Jun: fishing days related to June for trammel nets<br>*GTR_Jul: fishing days related to July for trammel nets<br>*GTR_Aug: fishing days related to August for trammel nets

n.	Dataset	File name	Brief description	Format	Licence	Temporal range of observations	Processing methodology	Number of records	Bounding box	Sea Area Common Name	CRS	Parameters
												*GTR_Sep: fishing days related to September for trammel nets<br>*GTR_Oct: fishing days related to October for trammel nets<br>*GTR_Nov: fishing days related to November for trammel nets<br>*GTR_Dec: fishing days related to December for trammel nets<br>*GTR_year: annual fishing days resulting from the sum of the monthly fishing days for trammel nets<br><br>*GTR_count: sum of the vessels (polygons) insisting on that cell for trammel nets<br>*GTR_amp: belonging area; it depends on the original polygons employing trammel nets, and, therefore, on where the interview was made (indeed, some cells may be located between two areas)<br><br>*GTR_spabb: species fished in that cell through trammel nets - abbreviated species names following the FAO nomenclature based on 3-Alpha Species Codes<br>*GTR_sp: species fished in that cell through trammel nets<br>*GTN_Jan: fishing days related to January for castellated/combined nets

n.	Dataset	File name	Brief description	Format	Licence	Temporal range of observations	Processing methodology	Number of records	Bounding box	Sea Area Common Name	CRS	Parameters
												*GTN_Feb: fishing days related to February for castellated/combined nets<br>*GTN_Mar: fishing days related to March for castellated/combined nets<br>*GTN_Apr: fishing days related to April for castellated/combined nets<br>*GTN_Mag: fishing days related to May for castellated/combined nets<br>*GTN_Jun: fishing days related to June for castellated/combined nets<br>*GTN_Jul: fishing days related to July for castellated/combined nets<br>*GTN_Aug: fishing days related to August for castellated/combined nets<br>*GTN_Sep: fishing days related to September for castellated/combined nets<br>*GTN_Oct: fishing days related to October for castellated/combined nets<br>*GTN_Nov: fishing days related to November for castellated/combined nets<br>*GTN_Dec: fishing days related to December for castellated/combined nets<br><br>*GTN_year: annual fishing days resulting from the sum of the monthly fishing days for castellated/combined nets

n.	Dataset	File name	Brief description	Format	Licence	Temporal range of observations	Processing methodology	Number of records	Bounding box	Sea Area Common Name	CRS	Parameters
												*GTN_count: sum of the vessels (polygons) insisting on that cell for castellated/combined nets<br><br>*GTN_amp: belonging area; it depends on the original polygons employing castellated/combined nets, and, therefore, on where the interview was made (indeed, some cells may be located between two areas)<br>*GTN_spabb: species fished in that cell through castellated/combined nets - abbreviated species names following the FAO nomenclature based on 3-Alpha Species Codes<br>*GTN_sp: species fished in that cell through castellated/combined nets<br>*GND_Jan: fishing days related to January for driftnets<br>*GND_Feb: fishing days related to February for driftnets<br><br>*GND_Mar: fishing days related to March for driftnets<br>*GND_Apr: fishing days related to April for driftnets<br>*GND_Mag: fishing days related to May for driftnets<br>*GND_Jun: fishing days related to June for driftnets<br>*GND_Jul: fishing days related to July for driftnets

n.	Dataset	File name	Brief description	Format	Licence	Temporal range of observations	Processing methodology	Number of records	Bounding box	Sea Area Common Name	CRS	Parameters
												*GND_Aug: fishing days related to August for driftnets<br>*GND_Sep: fishing days related to September for driftnets<br>*GND_Oct: fishing days related to October for driftnets<br>*GND_Nov: fishing days related to November for driftnets<br>*GND_Dec: fishing days related to December for driftnets<br>*GND_year: annual fishing days resulting from the sum of the monthly fishing days for driftnets<br>*GND_count: sum of the vessels (polygons) insisting on that cell for driftnets<br>*GND_amp: belonging area; it depends on the original polygons employing driftnets, and, therefore, on where the interview was made (indeed, some cells may be located between two areas)<br><br>*GND_spabb: species fished in that cell through driftnets - abbreviated species names following the FAO nomenclature based on 3-Alpha Species Codes<br><br>*GND_sp: species fished in that cell through driftnets

n.	Dataset	File name	Brief description	Format	Licence	Temporal range of observations	Processing methodology	Number of records	Bounding box	Sea Area Common Name	CRS	Parameters
												*LLS_Jan: fishing days related to January for set longlines<br>*LLS_Feb: fishing days related to February for set longlines<br>*LLS_Mar: fishing days related to March for set longlines<br>*LLS_Apr: fishing days related to April for set longlines<br>*LLS_Mag: fishing days related to May for set longlines<br>*LLS_Jun: fishing days related to June for set longlines<br>*LLS_Jul: fishing days related to July for set longlines<br>*LLS_Aug: fishing days related to August for set longlines<br>*LLS_Sep: fishing days related to September for set longlines<br>*LLS_Oct: fishing days related to October for set longlines<br>*LLS_Nov: fishing days related to November for set longlines<br>*LLS_Dec: fishing days related to December for set longlines<br>*LLS_year: annual fishing days resulting from the sum of the monthly fishing days for longlines

n.	Dataset	File name	Brief description	Format	Licence	Temporal range of observations	Processing methodology	Number of records	Bounding box	Sea Area Common Name	CRS	Parameters
												*LLS_count: sum of the vessels (polygons) insisting on that cell for longlines<br>*LLS_amp: belonging area; it depends on the original polygons employing longlines, and, therefore, on where the interview was made (indeed, some cells may be located between two areas)<br><br>*LLS_spabb: species fished in that cell through longlines - abbreviated species names following the FAO nomenclature based on 3-Alpha Species Codes<br>*LLS_sp: species fished in that cell through longlines<br>*Species_ab: same as "Species" field, but species are reported as 3-Alpha Species Codes following the FAO nomenclature<br>*Species: all the species fished in the considered cell, deriving from the overlapping of the different polygons (different gears used)<br>*MPA: belonging area; it depends on the original polygons and, therefore, on where the interview was made (indeed, some cells may be located between two areas)

n.	Dataset	File name	Brief description	Format	Licence	Temporal range of observations	Processing methodology	Number of records	Bounding box	Sea Area Common Name	CRS	Parameters
												*Gear: all the fishing gears employed within that cell, deriving from the overlapping of different polygons

Supplementary Table S3

Result of the classification of the selected fishing trips.
Of the 20567 selected fishing trips, 3352 were discarded from the fishing gear assignment process due to poor AIS signal quality.

**CLASSIFICATION - predicted fishing gear**	**Fishing trips**	**Percentage**
OTB	13009	76%
OTHER	1916	11%
PS	2290	13%
**Total**	**17215**	

Supplementary Table S4

Composition of the fleet active in GSA10 in 2019 broken down by type of license (primary - secondary) and nationality; segment of fleet reported in the EUFR and GFCM registers.Therefore, from the overlay of the AIS 2019 signal with the GSA10, 392 active vessels were identified, of which 58 correspond to vessels from the FEAMP register.
Excluding the latter, 334 boats were identified, of which 324 were associated with the registers (EUFR and GFCM) through the matching procedure.

	**License**	**VL(0612]**	**VL(1218]**	**VL(1824]**	**VL(2440]**	**VL(40XX)**
**Country**	PS-NA	0	0	0	3	5
France	DRB-PS	1	0	0	0	0
Italy	GNS-LTL	0	2	0	0	0
Italy	GNS-NO	0	1	0	0	0
Italy	LLS-GND	0	7	6	0	0
Italy	LLS-GNS	2	8	5	0	0
Italy	LLS-LHP	0	0	1	0	0
Italy	LLS-NO	0	0	1	0	0
Italy	LTL-NO	0	1	0	0	0
Italy	OTB-GNS	0	1	4	2	0
Italy	OTB-LLS	0	10	35	11	0
Italy	OTB-NO	0	13	38	44	0
Italy	OTB-PS	0	12	30	8	0
Italy	OTB-PTM	0	3	3	3	0
Italy	PS-GND	0	2	0	0	0
Italy	PS-GNS	0	2	1	0	1
Italy	PS-LHP	0	0	0	1	0
Italy	PS-LLS	1	16	17	1	0
Italy	PS-NO	0	0	4	4	2
Italy	PS-OTB	0	0	1	0	0
Italy	FPO-FPO	0	0	0	1	0
Malta	LLD-LLD	0	0	1	0	0
Malta	LLD-LLS	0	0	3	0	0
Malta	LLS-LLD	0	0	1	0	0
Malta	LLS-FPO	0	0	0	1	0
Portugal	GNS-GTR	0	0	0	2	0
Spain	PS1-NA	0	0	0	1	1
Turkey		4	78	151	82	9
	**Total**	**324**				

Supplementary Table S5

Composition of the FEAMP fleet active in 2019 according to the fishinglicenses (primary and secondary) and fleet segments reported in the project register.
From the FEAMP register, 60 boats present in the AIS data were identified, of which 2 were inactive in 2019.

	N°. of fishing vessels			
**License**	**VL(1218]**	**VL(1824]**	**VL(2440]**	**VL(40XX)**
OTB-GND	0	1	0	0
OTB-GNS	0	1	0	0
OTB-LLS	2	3	0	0
OTB-NO	7	10	4	0
OTB-PS	0	1	2	0
OTB-PTM	0	2	0	0
PS-GNS	2	2	0	0
PS-LLS	1	1	2	3
PS-NO	0	5	0	3
PS-OTB	0	0	0	2
PS-PS	0	0	2	4
**Total**	**12**	**26**	**10**	**12**

Supplementary Table S6

Table recording the 51 encountered target species.
Here the Latin name, a shortened version of the Latin name, the English common name and the 3-Alpha Species Code nomenclature proposed by FAO have been provided.

n.	Latin name	Latin name - shortened version	English common name	3-Alpha Species Code (FAO nomenclature)
1	Merluccius merluccius	M. merluccius	European hake	HKE
2	Scorpaena spp	Scorpaena spp	Rockfish	SCS
3	Palinurus elephas	P. elephas	Common spiny lobster	SLO
4	Mullus spp	Mullus spp	Surmullet	MUX
5	Octopus vulgaris	O. vulgaris	Common octopus	OCC
6	Sepia officinalis	S. officinalis	Common cuttlefish	CTC
7	Scomber spp	Scomber spp	Scomber mackerel	MAZ
8	Illex spp	Illex spp	Shortfin squid	ILL
9	Diplodus spp	Diplodus spp	Sargo bream	SRG
10	Seriola dumerili	S. dumerili	Greater amberjack	AMB
11	Sarpa salpa	S. salpa	Salerna	SLM
12	Mugilidae	Mugilidae	Mullet	MUL
13	Chelidonichthys spp	Chelidonichthys spp	Indopacific gurnard	GUI
14	Other	Other	Other	Other
15	Solea spp	Solea spp	Sole	SOO
16	Lophius spp	Lophius spp	Monkfish	MNZ
17	Sarda sarda	S. sarda	Atlantic bonito	BON
18	Euthynnus alletteratus	E. alletteratus	Little tunny	LTA
19	Coryphaena hippurus	C. hippurus	Common dolphinfish	DOL
20	Scorpaena porcus	S. porcus	Black scorpionfish	BBS
21	Sciaena umbra	S. umbra	Brown meagre	CBM
22	Pagellus spp	Pagellus spp	Pandora	PAX
23	Rajidae	Rajidae	Rays	RAJ
24	Loligo vulgaris	L. vulgaris	European squid	SQR
25	Muraena helena	M. helena	Mediterranean moray	MMH
26	Conger conger	C. conger	European conger	COE
27	Arnoglossus laterna	A. laterna	Mediterranean scaldfish	MSF
28	Pagellus erythrinus	P. erythrinus	Common pandora	PAC
29	Dentex dentex	D. dentex	Common dentex	DEC
30	Epinephelus spp	Epinephelus spp	Grouper	GPX
31	Pleuronectes platessa	P. platessa	European plaice	PLE
32	Lepidopus caudatus	L. caudatus	Silver scabbardfish	SFS
33	Pagellus bogaraveo	P. bogaraveo	Blackspot seabream	SBR
34	Zeus faber	Z. faber	John dory	JOD
35	Mullus barbatus	M. barbatus	Red mullet	MUT
36	Scophthalmidae	Scophthalmidae	Turbot	SCF
37	Uranoscopus scaber	U. scaber	Stargazer	UUC
38	Lithognathus mormyrus	L. mormyrus	Sand steenbras	SSB
39	Sparus aurata	S. aurata	Gilthead seabream	SBG

n.	Latin name	Latin name - shortened version	English common name	3-Alpha Species Code (FAO nomenclature)
40	Trachurus spp	Trachurus spp	Jack and horse mackerel	JAX
41	Engraulis encrasicolus	E. encrasicolus	European anchovy	ANE
42	Phycis spp	Phycis spp	Forkbeard	FOX
43	Lichia amia	L. amia	Leerfish	LEE
44	Sphyraena spp	Sphyraena spp	Barracuda	BAR
45	Pagellus acarne	P. acarne	Axillary seabream	SBA
46	Xyrichtys novacula	X. novacula	Pearly razorfish	XYN
47	Auxis rochei	A. rochei	Bullet tuna	BLT
48	Nephrops norvegicus	N. norvegicus	Norway lobster	NEP
49	Aristaeomorpha foliacea	A. foliacea	Giant red shrimp	ARS
50	Spondyliosoma cantharus	S. cantharus	Black seabream	BRB
51	Pagrus pagrus	P. pagrus	Red porgy	RPG
